# Tubular Structure Segmentation via Multi-Scale Reverse Attention Sparse Convolution

**DOI:** 10.3390/diagnostics13132161

**Published:** 2023-06-25

**Authors:** Xueqiang Zeng, Yingwei Guo, Asim Zaman, Haseeb Hassan, Jiaxi Lu, Jiaxuan Xu, Huihui Yang, Xiaoqiang Miao, Anbo Cao, Yingjian Yang, Rongchang Chen, Yan Kang

**Affiliations:** 1 School of Applied Technology, Shenzhen University, Shenzhen 518060, China; 2100411016@stumail.sztu.edu.cn (X.Z.); 2070416007@stumail.sztu.edu.cn (J.L.); 2210417016@stumail.sztu.edu.cn (H.Y.); 2210417004@stumail.sztu.edu.cn (A.C.); 2 College of Health Science and Environmental Engineering, Shenzhen Technology University, Shenzhen 518118, China; 1910442@stu.neu.edu.cn (Y.G.); zamanasim2021@email.szu.edu.cn (A.Z.); haseeb@sztu.edu.cn (H.H.); 2271341@stu.neu.edu.cn (X.M.); 1810453@stu.neu.edu.cn (Y.Y.); 3 College of Medicine and Biological Information Engineering, Northeastern University, Shenyang 110169, China; 4 School of Biomedical Engineering, Medical School, Shenzhen University, Shenzhen 518060, China; 5 State Key Laboratory of Respiratory Disease, National Center for Respiratory Medicine, National Clinical Research Center for Respiratory Disease, First Affiliated Hospital, Guangzhou Medical University, Guangzhou 510120, China; xujiaxuan@stu.gzhmu.edu.cn; 6 Shenzhen Institute of Respiratory Diseases, Shenzhen People’s Hospital, Shenzhen 518001, China; 7 The Second Clinical Medical College, Jinan University, Guangzhou 518001, China; 8 The First Affiliated Hospital, Southern University of Science and Technology, Shenzhen 518001, China; 9 Engineering Research Centre of Medical Imaging and Intelligent Analysis, Ministry of Education, Shenyang 110169, China

**Keywords:** cerebrovascular, airway, tubular structures, multi-scale, reverse attention, sparse convolution

## Abstract

Cerebrovascular and airway structures are tubular structures used for transporting blood and gases, respectively, providing essential support for the normal activities of the human body. Accurately segmenting these tubular structures is the basis of morphology research and pathological detection. Nevertheless, accurately segmenting these structures from images presents great challenges due to their complex morphological and topological characteristics. To address this challenge, this paper proposes a framework UARAI based on the U-Net multi-scale reverse attention network and sparse convolution network. The framework utilizes a multi-scale structure to effectively extract the global and deep detail features of vessels and airways. Further, it enhances the extraction ability of fine-edged features by a joint reverse attention module. In addition, the sparse convolution structure is introduced to improve the features’ expression ability without increasing the model’s complexity. Finally, the proposed training sample cropping strategy reduces the influence of block boundaries on the accuracy of tubular structure segmentation. The experimental findings demonstrate that the UARAI-based metrics, namely Dice and IoU, achieve impressive scores of 90.31% and 82.33% for cerebrovascular segmentation and 93.34% and 87.51% for airway segmentation, respectively. Compared to commonly employed segmentation techniques, the proposed method exhibits remarkable accuracy and robustness in delineating tubular structures such as cerebrovascular and airway structures. These results hold significant promise in facilitating medical image analysis and clinical diagnosis, offering invaluable support to healthcare professionals.

## 1. Introduction

Cerebrovascular and airway structures are vital tubular structures in the human body that play key roles in brain blood transport and respiratory gas exchange, respectively. Cerebrovascular structures provide blood and oxygen to the brain tissues, and once cerebrovascular pathologies occur, they can seriously affect brain tissue function [1]. Airway structures are responsible for the exchange of gases between the human body and the outside world, but they are easily affected by toxic air pollution, leading to diseases that can develop into respiratory tract diseases. In recent years, the incidence of cerebrovascular and lung respiratory tract diseases has been increasing year by year, which has caused serious impacts on patients, society, and the nation [2]. Studies have shown that cerebrovascular and respiratory tract diseases are closely related to the morphological changes of cerebrovascular and airway structures [3]. Therefore, segmentation of tubular structures, including cerebrovascular and airway structures, can help understand the distribution of morphological structures and support the diagnosis and detection of relevant diseases. In conclusion, the segmentation of cerebrovascular and airway structures is a crucial foundation for the research, analysis, and identification of cerebrovascular and respiratory system diseases, which carries critical implications for both clinical practice and academic research.

Currently, medical imaging is the primary means to investigate the morphology of cerebrovascular and airway structures. For instance, magnetic resonance angiography (MRA) is a widely used non-ionizing radiation and contrast-agent-free imaging technique for studying cerebrovascular diseases [4]. The main imaging methods of MRA include time-of-flight (TOF), phase contrast (PC), fresh blood imaging (FBI), and contrast-enhanced MRA [5]. Among these methods, TOF-MRA is the most commonly used imaging method in non-invasive vascular studies and is widely used in clinics due to its fast imaging speed and high contrast [6]. Unlike cerebrovascular structures, airway structures are scanned by computed tomography (CT) equipment. It has the advantages of high resolution, rich grayscale information, and convenient acquisition, making it widely used in airway segmentation. The segmentation of cerebrovascular and airway trees is a significant challenge in the medical field due to their complex three-dimensional structures, which have varying lengths, widths, and distributions. As a result of these complexities, their segmentation remains a research priority. Segmentation methods for tubular structures are mainly divided into manual, semi-automatic, and automatic segmentation. Manual segmentation requires a large amount of time and effort [7]. According to statistics, the average annotation time for each patient’s cerebrovascular data is about 60–80 min [8], and manual scanning and labeling of CT images requires more than 15 h [9]. Semi-automatic and automatic segmentation methods have significantly improved efficiency compared with manual segmentation. Implementing automatic segmentation methods can significantly reduce the errors and inconsistencies caused by human factors, improving the overall accuracy and stability of segmentation. Semi-automatic scanning and labeling of airway trees take about 2.5 h [10], while automatic segmentation using a pre-trained model can be completed in only 2–3 min. With the rapid development of science and technology, numerous automatic segmentation techniques for blood vessels and airways have been proposed, aiming to improve segmentation accuracy, assist clinical diagnosis of relevant diseases, reduce doctors’ workload, improve work efficiency, and promote the development of computer-aided medicine [11].

In recent years, many deep-learning-based methods for 3D medical image segmentation have been proposed, and have shown promising results. Most of them follow the encoder-decoder architecture similar to U-Net [12], and 3D U-Net [13] was initially introduced with excellent performance. Later, the topology of U-Net proposed by Tetteh et al. [14], Livne et al. [15], Lee et al. [16], Hilbert et al. [8], and Oktay et al. [17] has been used for 3D medical segmentation, especially for tubular structure segmentation. However, due to the complexity and diverse morphology of branch-like tubular structures, such as brain vessels and airways, and the severe imbalance between target voxels and other voxels during the segmentation process, some methods do not achieve high segmentation accuracy. While it is relatively easy to differentiate between coarse vessels and the background in TOF-MRA images, the segmentation of small vessels and edge details often tends to be inadequate. Similarly, segmenting small airways in CT lung images is also very challenging [6,18]. Improving edge segmentation of such tubular structures is key to subsequent quantification of vessels and airway structures. In summary, improving the segmentation accuracy of branch-like tubular structures such as brain vessels and airways is very important, and enhancing their branch edge segmentation accuracy is crucial to improving the segmentation of tubular structures.

To address this limitation, this paper proposes a deep learning network UARAI (U-Net multi-scale feature aggregation reverse attention sparse convolution model) for segmenting complex tubular structures. The model comprehensively considers the structural characteristics of tubular structures at different scales, enhances the learning of features such as edge details, micro-vessels, and micro-airways, and aims to improve the segmentation accuracy. The main contributions of this work are summarized as follows:

(a) In this paper, a multi-scale feature aggregation method is proposed and validated, which can fully extract and fuse the cerebrovascular and airway features with different thicknesses at different scales. The proposed method effectively solves the problem of differences in feature expression at the same scale, thus improving the segmentation accuracy.
(b) Our paper introduces a novel reverse attention module combined with sparse convolution to guide the network effectively. By leveraging reverse attention mechanisms, this module enhances foreground detection by emphasizing the background and excluding areas of prediction. Moreover, it allocates reverse attention weights to extracted features, thereby improving the representation of micro-airways, micro-vessels, and image edges. The utilization of sparse convolution further improves overall feature representation and segmentation accuracy.
(c) Through extensive experimental validation, we investigate the impact of sliding window sequencing and input image dimensions on the segmentation of tubular structures, including cerebral blood vessels and airways. The insights gained from this study contribute to the advancement of artificial intelligence techniques in medical image analysis, specifically focusing on enhancing the segmentation of tubular structures.

## 2. Related Work

In this section, we briefly review the related work and start-of-the-art approaches for tubular structure segmentation, feature fusion and 3D attention mechanisms for medical images.

### 2.1. Tubular Structure Segmentation

In medical image processing, the methods for segmenting tubular structures can be mainly classified into two categories: traditional methods and deep-learning-based methods.

Traditional methods: Traditional medical image segmentation methods originate in traditional imaging techniques, primarily relying on image gray features for segmentation. For cerebrovascular segmentation, Park et al. [19] proposed a connectivity-based local adaptive threshold algorithm for carotid artery segmentation. The algorithm adaptively segments the cerebrovascular structures based on the connectivity preserved between consecutive slices of the image and the local threshold set on each slice. Wang et al. [20,21,22] used Ostu’s threshold to classify MRA images into foreground and background and then compared the statistical distributions of foreground and background to extract cerebrovascular structures from the foreground. Neumann et al. [23] combined vessel-enhanced filtering with subsequent level set segmentation, where level set segmentation was implemented using gradient descent and local minimum energy functions. Subsequent studies have also proposed other level set segmentation methods. Still, since they are susceptible to grayscale values and significantly impact the algorithm’s convergence, the problem of segmentation difficulty remains [24]. Subsequently, Frangi et al. [25] proposed a Hessian-matrix-based method, known as the Frangi algorithm, which calculates the local Hessian-matrix of each pixel in an image to determine the vascular structure’s location precisely. This approach has been shown to significantly enhance the performance of vessel segmentation compared to traditional segmentation methods. For airway segmentation, early works by Mori and Sonka et al. [26,27] used the difference in grayscale intensity between airway lumen and wall, combined with region-growing algorithms, for airway lumen segmentation. Tschirren et al. [28] proposed a fuzzy connectivity-based airway segmentation method that uses small adaptive regions to follow the airway branching. Duan et al. [29] proposed combining a dual-channel region-growing algorithm, grayscale morphological reconstruction, and leakage elimination. The method first performs the region-growing on one channel to obtain a rough airway tree, then does region-growing and grayscale morphological reconstruction on another channel to detect distant airways, and finally refines the airway tree by removing holes and leaks using the leakage detection method. While traditional methods can somewhat segment tubular structures, image quality and differences in imaging parameters often influence their performance. For instance, threshold segmentation algorithms can efficiently segment foreground and background but have difficulty distinguishing appropriate thresholds for noise with comparable grayscale values as the target object [30]. Additionally, the grayscale intensity of cerebrovascular and airway branches resembles the background, and their peripheral structures are intricate and complicated. As a result, traditional threshold segmentation and region-growing methods often struggle to achieve precise segmentation.

Deep-learning-based methods: In recent years, medical image segmentation has benefited from applying artificial intelligence (AI) technologies. Among these, deep-learning techniques are considered the most sophisticated and commonly used techniques [31]. The neuro-heuristic [32] analysis algorithm has made significant advances in the field of medical image segmentation by providing a deeper analysis of images for classification, segmentation, and recognition. However, it requires a large volume of high-quality image data for processing medical image segmentation tasks. Additionally, the complex network design of neuro-heuristic analysis algorithms and their empirical nature result in lower interpretability compared to traditional machine-learning algorithms. The Fox algorithm [33] has performed well in lung segmentation of medical images by automatically learning specific features such as lung position and shape for more accurate and efficient segmentation results. However, the Fox algorithm requires a large amount of training data and consumes considerable computational resources and time, resulting in lower segmentation efficiency. However, among the existing deep learning networks, U-Net is widely used in medical image segmentation tasks with scarce labeled data due to its small data requirement and fast training speed [12]. In the cerebrovascular segmentation task, Tetteh et al. [14] provided synthesized brain vessel tree data and used it for transfer learning to achieve efficient, robust, and universal vessel segmentation. Livne et al. [15] used a 2D U-Net network to segment cerebrovascular structures in high quality and compared Half-U-Net with half-channel numbers and found that Half-U-Net had equally excellent evaluation performance indices as U-Net. Lee et al. [16] proposed the Spider U-Net, which is based on the U-Net structure and enhances the connectivity of blood vessels between axial slices by inserting long short-term memory (LSTM) into the baseline model. At the same time, using the striding stencil (SS) data transfer strategy greatly improved the brain vessel segmentation effect. Guo et al. [11] proposed the M-U-Net model, which consists of three 2D U-Nets and fuses image features in three directions, inheriting the excellent performance of 2D U-Net in image segmentation and making up for the deficiency of a single U-Net in extracting 3D image axial features. Cicek et al. [13] designed a 3D U-Net segmentation network based on 2D U-Net, incorporating image z-axis information to improve segmentation accuracy. Hilbert et al. [8] proposed a high-performance, fully automatic segmentation framework BRAVE-NET, combining deep supervised networks and aggregating rough and low-resolution feature maps into the final convolution layer, effectively fusing multi-scale features. Min et al. [34] introduced multi-scale inputs and residual mechanisms into the U-Net network to improve the model’s performance while maintaining generalization ability. Oktay et al. [17] introduced a novel module known as the Self-Attention Gate module, which enhances the significance of local regions and improves the model’s sensitivity to the foreground, ultimately enhancing segmentation accuracy. Mou et al. [35] introduced the CS2-Net network structure for automatic detection of curved structures in medical and biomedical images. They incorporated self-attention mechanisms in both the encoder and decoder to enhance the features of curved structures. Xia et al. [36] proposed a reverse edge attention module and an edge-enhanced optimized loss to emphasize the importance of voxels along 3D body edges. Their approach aimed to better capture and preserve spatial edge information. Chen et al. [37] developed an attention and generative adversarial network model for brain vessel segmentation. They utilized multilevel features and dense connections to establish local and global associations. Additionally, they incorporated attention mechanisms in the discriminator to filter low-level features, balance the proportion of vessel class, and improve segmentation performance. Banerjee et al. [38] introduced the multi-task deep CNN (MSD-CNN) approach, which learns the voxel-wise centrality of the surface of cerebral vessels. This method adds additional regularization to the segmentation task. Jiang et al. [39] proposed the Axis-Projection Attention Network (APA U-Net) for 3D medical image segmentation, with a specific focus on small-object segmentation. The network employs a projection strategy that projects 3D features onto three orthogonal 2D planes to capture contextual attention from different viewpoints. This enables the network to filter out redundant feature information and retain crucial details of small lesions in 3D scans. For the airway segmentation task, Meng et al. [40] presented a method that combines 3D deep learning with image-based tracking to automatically extract airways. They employed adaptive cube volume analysis based on 3D U-Net models, where the 3D U-Net is used to extract the airway region within the volume of interest (VOI) for precise airway segmentation. Garcia Uceda et al. [41] used various data augmentation methods based on the 3D U-Net network to achieve accurate airway segmentation, and Garcia Uceda et al. [42] proposed another method combining 3D U-Net with graph neural networks, which uses graph convolution layers instead of ordinary convolution layers, achieving accurate airway tree segmentation with fewer training parameters. Wang et al. [43] used U-Net with spatial recurrent convolutional layers and radial distance loss function (RD Loss) to better segment tubular structures. Tan et al. [44] compared the methods of 12 teams in the airway segmentation challenge task at the 4th International Symposium on Image Computing and Digital Medicine (ISICDM 2020) and found that nine teams adopted U-Net networks or other forms of U-Net, including the forward attention mechanism, reverse attention mechanism, and multi-scale feature information fusion structure, and analyzed the effect of different networks on airway segmentation.

### 2.2. Multi-Scale Feature Fusion and Attention Mechaism

In medical image segmentation, feature fusion combines multiple heterogeneous features into a feature with high discriminative ability, improving the segmentation accuracy. In medical image segmentation networks, low-level feature layers have a high resolution and contain rich primary features, such as position, shape, and texture information. High-level feature layers have strong semantic information and a large receptive field, but low resolution and poor perception of details. Lin et al. [45] proposed the feature pyramid networks (FPN) that complemented different levels of feature maps, generating a feature map that simultaneously possesses high resolution and deep-level information. FPN allows the various levels of feature maps to complement each other, forming a multi-scale feature map by adding special lateral connections during the up-sampling and down-sampling process. This feature map can be used to detect objects of different sizes, solving the difficulty of multi-scale object detection. It effectively utilizes different feature maps of different scales to detect objects of different sizes, improving the accuracy of object detection. He et al. [46] proposed a Spatial Pyramid Pooling (SPP) structure that can handle input images at different scales, effectively addressing the problem of varying input image sizes and improving the network’s classification performance. Zhao et al. [47] proposed the Pyramid Scene Parsing Network (PSP-Net), which uses dilated convolution to process context features of different regions to obtain global context information features, solving the utilization of global context features and multi-scale feature processing in semantic segmentation. Chen et al. [48] proposed the Atrous Spatial Pyramid Pooling (ASPP) module, which combined multiple feature maps with different resolutions obtained by dilated convolutions to obtain a feature map with a global receptive field, significantly improving the performance of image segmentation. In summary, fusing features at different scales is an important means of improving segmentation performance.

The introduction of the attention mechanism has greatly improved feature selection ability in many computer vision tasks. Similarly, it has been widely applied in medical image segmentation. Tran introduced spatial attention in convolutional neural networks, allowing the network model to learn features from different regions of the image more accurately. Later, the spatial attention mechanism was gradually applied to medical image analysis, achieving good results. Hu et al. [49] proposed an SE (Squeeze-and-Excitation) mechanism to weight and rescale feature maps using the importance ratio of each channel, which is widely used for medical image segmentation and classification. Woo further improved the model performance by adding the spatial attention mechanism based on SE attention. In early CT abdominal vessel segmentation, Oktay et al. [17] proposed a network structure with attention gates, enabling the network to automatically focus on organ structures in the image. Fan et al. [50] proposed a reverse attention U-Net structure for polyp segmentation, in which the reverse attention (RA) module implicitly erases the predicted region and highlights the background, guiding the network to gradually explore the polyp region and enhance the edge feature learning, improving segmentation accuracy. Mou et al. [35] designed a CS2-Net for detecting curved structures in medical images, such as blood vessels, by introducing self-attention, spatial attention (SAB) and channel attention (CAB). Xia et al. [36] proposed a reverse attention mechanism for edge enhancement features and introduced an edge-reinforced loss for vascular shape segmentation. While various attention mechanisms can effectively enhance feature representativeness, challenges still exist in edge segmentation of complex structures and microstructures.

## 3. Materials and Methods

### 3.1. Materials

#### 3.1.1. Datasets

This article demonstrates the wide applicability of UARAI in 3D tubular structure segmentation by validating public cerebrovascular data and airway tree data (as shown in Table 1) provided by cooperating organizations. The cerebrovascular dataset comes from the open dataset MIDAS [51], which contains MRA images of 109 healthy volunteers aged 18 to over 60. These images were acquired by a standardized protocol 3T MRI scanner with a voxel size of 0.5 mm × 0.5 mm × 0.8 mm and a uniform sampling resolution of 448 × 448 × 128. The segmentation labels were initially annotated using 3Dslicer and ITK-Snap software (Version.3.8.0). Subsequently, two professional doctors manually corrected and labeled each piece of cerebrovascular data to create a binary image with labels. In this image, the background is represented as 0, while the blood vessels are represented as 1.

The airway dataset consists of 400 samples obtained from lung CT data provided by Guangzhou Medical University. After excluding images of poor quality, a total of 380 samples were used for experimentation. The voxel size of the images was 0.67 mm × 0.67 mm × 1 mm, and the scanning resolution was uniformly resampled to 512 × 512 × 320. The labeled images were generated through interactive annotation conducted by three professional radiologists.

#### 3.1.2. Data Pre-Processing and Sample Cropping

**Figure 1:**
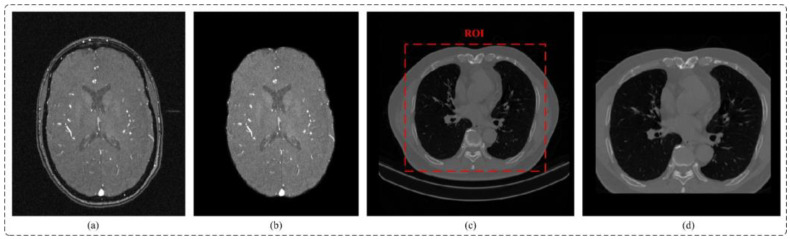
(**a**) TOF-MRA image (**b**) TOF-MRA after skull removal (**c**) CT image (**d**) Reduction of non-parenchymal regions.

Data pre-processing: TOF-MRA images collected by hospitals typically include the skull. Since the grayscale values of the skull and blood vessels are similar, the neural network may extract interference features from the skull when extracting brain vascular features. As a result, it is necessary to remove the skull. This study utilized FSL [52] and HD-Bet [53] tools to extract the brain region effectively, as depicted in Figure 1b. To diversify sample trends for brain vascular data, data augmentation methods such as random flipping, random affine, and elastic deformation were employed [42]. In the case of the lung CT dataset, non-pulmonary regions were eliminated by using data augmentation techniques such as cropping, random flipping, and rotation, as shown in Figure 1d. Since numerical values are large and pixel distribution is scattered for the MRA and lung CT images, Z-score normalization was used. The advantages include a reduction of computational complexity, increased utilization of computer resources, and improved convergence rate and efficiency of the network.

(1) $$x_{out}=\frac{x_{in}-mean(x_{in})}{std(x_{in})}$$

Here, $x_{in}$, $mean(x_{in})$, $std(x_{in})$, and $x_{out}$ respectively represent the input image, the mean of input image grayscale, the variance of input image grayscale, and the normalized output image.

Training sample cropping: In the field of medical imaging, image categories such as MRI, pathological images, and 3D CT images often have large file sizes. Directly training models on these images can be unrealistic and inefficient [54,55]. Therefore, this study utilized high-resolution 3D TOF-MRA images and 3D lung CT images for training, using image patches to train the model. This increases the number of training samples and reduces the GPU memory costs for model training. To extract the patches, we used a sliding window approach combined with random cropping. In addition, the size of the patch is an important factor affecting the model’s performance [56]. We set the patch size to 64 × 64 × 32, with a cross-sectional size of 64 × 64. Since the MRA image has a small scale on the z-axis, the size of the z-axis was set to 32. The patch z-axis for lung airway data was also set to 32 to ensure consistent training parameters. During the prediction phase, we also used a sliding window prediction strategy, predicting individual patches one by one and then stitching the predicted results back to the original image size to obtain the segmentation results.

### 3.2. Methods

#### UARAI Overall Framework

**Figure 2:**
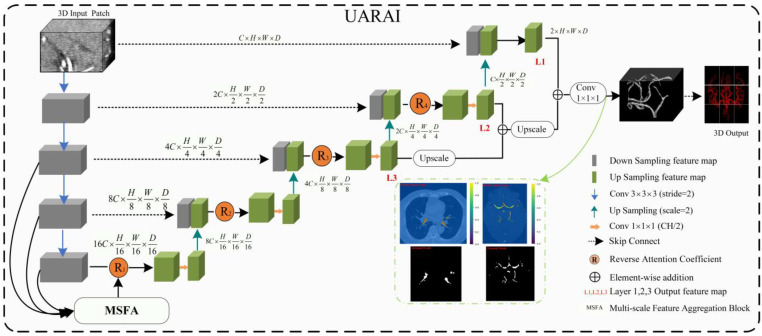
The overall network framework of the UARAI network. The overall architecture is constructed based on the 3D U-Net. Firstly, at the encoding stage, the multi-scale feature aggregation module (MSFA) is applied to integrate features from different scales. In addition, a reverse attention module is incorporated after the jump connection to calculate the reverse attention coefficients. The coefficients are then used to re-weight the foreground and thus enhance the feature expression ability.

The U-Net framework has been widely applied in medical image segmentation and is considered one of the most promising frameworks [12]. In this paper, we propose a novel network framework, UARAI, based on the 3D U-Net architecture and integrates advanced techniques, such as multi-scale feature aggregation, reverse attention, and inception sparse convolution structure. This framework can achieve high-precision automatic segmentation of the cerebrovascular and airway structures. The network input is a cerebrovascular patch $x\in P^{1\times H\times W\times D}$, where *H*, *W*, and *D* represent length, width, and depth, respectively. The output of UARAI predicts foreground and background segmentation probability maps $y\in P^{2\times H\times W\times D}$, with the specific network structure illustrated in Figure 2.

The proposed UARAI segmentation network is based on the 3D U-Net framework. The encoder is achieved for image down-sampling and multi-scale feature extraction, while the decoder reconstructs high-resolution feature maps through up-sampling and skip connections. Each layer in the encoder path consists of multiple convolutional layers for feature extraction. Furthermore, this network utilizes a stride-2 convolutional layer, which learns the parameters of convolutional kernels, to increase the network’s representation ability and achieve dimensionality reduction of features instead of a pooling layer. Additionally, the lack of shallow critical features can somewhat affect the segmentation results due to the loss of some low-level features during the dimensionality reduction process in the encoder path. Considering the uneven thickness of blood vessels and airways, and the differences in the expression of coarse tube-like structures at different scales, this paper adds a multi-scale feature aggregation module (MSFA) to the encoder path. This module aggregates shallow and deep features at different scales to help the network learn features better at different scales and improve feature extraction ability.

In the decoder path, the integration of low-level and high-level features is achieved by utilizing skip connections to combine the encoded feature map with the decoded feature map. This process ensures a comprehensive integration of information at different levels. Moreover, for accurate segmentation of small and intricate target branches such as blood vessels and airways, the inclusion of edge information is crucial. To address this, a reverse attention module (RAM) is incorporated into the decoder path. The RAM enhances the extraction and expression of edge features in the terminal branches. By multiplying the reverse attention coefficient with the feature map after the skip connection, the RAM dynamically adjusts the weight of the edge features. This adaptation aims to improve the accuracy of edge segmentation.

During the network output stage, the final three layers of the decoded output undergo operations such as up-sampling and convolutional fusion. These operations refine the feature maps and ultimately generate the segmentation results, which includes both foreground and background information as $y\in P^{2\times H\times W\times D}$.

The overall implementation process of the segmentation model is as follows: in the encoding phase, the input is a batch of patches. Each layer first extracts patch features through two convolutional modules and then reduces the dimension of the features through a learnable convolutional layer with a kernel size of 3 × 3 × 3 and a stride of 2 instead of a pooling operation. The features are then normalized and activated non-linearly through InstanceNorm3d and Leaky-Relu, producing non-linear features. Residual processing is also added in each layer to prevent excessive feature loss and gradient disappearance. In the decoding process, skip connections are first used to concatenate the encoding layer features with the decoding layer features. Then, reverse attention modules are used to reassign feature weights, adaptively enhance edge features, and obtain decoding layer features through convolution. Up-sampling is performed through interpolation to reach the next decoding stage. The encoding and decoding operations are repeated four times each, resulting in segmentation results of the same size as the original image. Finally, the soft-max function normalizes the probability of foreground and background in the output.

(A) Multi-Scale Feature Aggregation

Feature aggregation is commonly used in the field of computer vision. With the development of medical imaging, multi-scale feature aggregation has also been widely used in deep learning for medical image processing [57]. In the feature aggregation process, convolution, up-sampling, concatenation, and addition operations are used to fuse shallow and deep features, resulting in deep features that contain both strong expressions of high-level features with large receptive fields and rough features that represent edges and shapes in shallow layers. For example, considering the instance segmentation path aggregation network proposed by Liu et al. [58] has fully demonstrated the advantages of aggregating features at multiple levels for accurate prediction. On the other hand, in our approach, multi-scale feature aggregation is used to aggregate features of different scales obtained during the down-sampling process to the deep layers of the network to achieve full integration of high-level and shallow features.

**Figure 3:**
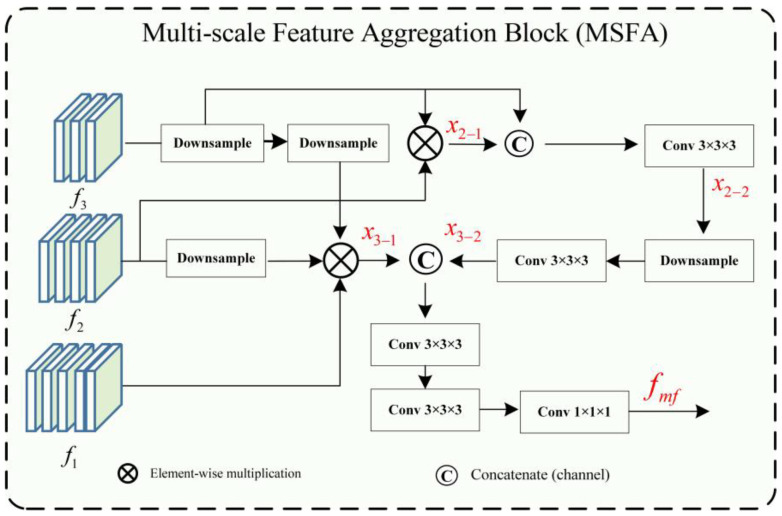
Structural diagram of the multi-scale feature aggregation module.

Cerebrovascular and airway structures both have the anatomical characteristics of complex branching and uneven thickness. Accurate segmentation of tube-like structures with varying thicknesses at high precision within the same scale is challenging. In general networks, features of different scales have different expression abilities for structures of different sizes and shapes. In our segmentation task, both the large targets (such as major vessels and main airways) and small targets (such as peripheral branches of vessels and airways) are equally important, and the absence of any feature can significantly impact segmentation accuracy and clinical diagnosis. Therefore, multi-scale feature aggregation is used to avoid the loss of these features. As shown in Figure 3, in the multi-scale feature aggregation framework, the input features represent the low- and high-level output features of the encoding layers of U-Net. The low-level feature maps mainly contain edge and texture information of the image, while the high-level features represent the semantic features with strong expression characteristics of the image. The lower-level features $f_{3}\in P^{4C\times \frac{H}{4}\times \frac{W}{4}\times \frac{D}{4}}$ are down-sampled to reduce their size by half through dimensionality reduction. Multiplying it with $f_{2}\in P^{8C\times \frac{H}{8}\times \frac{W}{8}\times \frac{D}{8}}$ produces $x_{2\_1}\in P^{8C\times \frac{H}{8}\times \frac{W}{8}\times \frac{D}{8}}$, which is concatenated with another down-sampled feature and fused through channel-wise concatenation, then the feature $x_{2\_2}\in P^{8C\times \frac{H}{8}\times \frac{W}{8}\times \frac{D}{8}}$ is extracted through Conv3 × 3 × 3 convolution. After that, $x_{2\_2}$ is down-sampled and passed through Conv3 × 3 × 3 convolution again to obtain $x_{3\_2}\in P^{16C\times \frac{H}{16}\times \frac{W}{16}\times \frac{D}{16}}$. Then, $f_{1}$ is multiplied with a down-sampled $f_{2}$ and $f_{3}$ down-sampled twice to obtain $x_{3\_1}\in P^{16C\times \frac{H}{16}\times \frac{W}{16}\times \frac{D}{16}}$. Finally, $x_{3\_1}$ and $x_{3\_2}$ are concatenated through channel-wise concatenation and passed through two Conv3 × 3 × 3 convolutions to extract features. The feature fusion and output are achieved through Conv1 × 1 × 1 to obtain $f_{mf}$. The utilization of a multi-scale feature fusion approach serves to enhance both global and intricate features significantly. By amalgamating features from diverse levels, a more comprehensive and expressive feature representation is achieved, leading to notable improvements in segmentation accuracy.

(2) $$f_{i}=E(image)$$

(3) $$\begin{array}{l} x_{2\_1}=D(f_{3})*f_{2}\\ x_{2\_2}=Conv[C(x_{2\_1},f_{3})]\\ x_{3\_1}=D^{2}(f_{3})*D(f_{2})*f_{1}\\ x_{3\_2}=Conv_{3\times 3\times 3}[D(x_{2\_2})] \end{array}$$

(4) $$f_{mf}=Conv_{1\times 1\times 1}\{Conv_{{}_{3\times 3\times 3}}^{2}[C(x_{3\_1},x_{3\_2})]\}$$

Here, image, *, E, D, C, $f_{i}$, and $f_{mf}$ represent the input image patch, the functions of matrix multiplication, encoder, down-sample, concatenate, encoder layer feature, and fused feature, respectively.

(B) Reverse Attention Block

The complex shapes, varied branching structures of normal and abnormal cerebrovascular and airway structures, inconsistent imaging intensity, and substantial inter-individual differences affect the segmentation of tubular structures. This is especially the case with the extraction of peripheral, edge, and detail features. The reverse attention mechanism proposed by Fan et al. [50] performs well in segmenting the edges of polyps. By repeatedly utilizing the Reverse Attention (Rattention) module, a relationship between regional and boundary clues can be established to extract edge features from the fused high-level features. Through continuous training iterations, the model can correct partially inconsistent areas in the predicted results, improving the segmentation accuracy.

In the network architecture proposed in this paper, the encoded features obtained are fused by skip connections to combine low-level and high-level features. However, the fused feature maps are not sensitive to edge details and edge features, which are difficult to extract from vessels and airways due to their rich branching structures and fine peripheral features. By multiplying the reverse attention coefficient matrix with the input features, the fused feature maps can be adaptively assigned with corresponding reverse attention weights, enhancing the expression of edge features.

**Figure 4:**
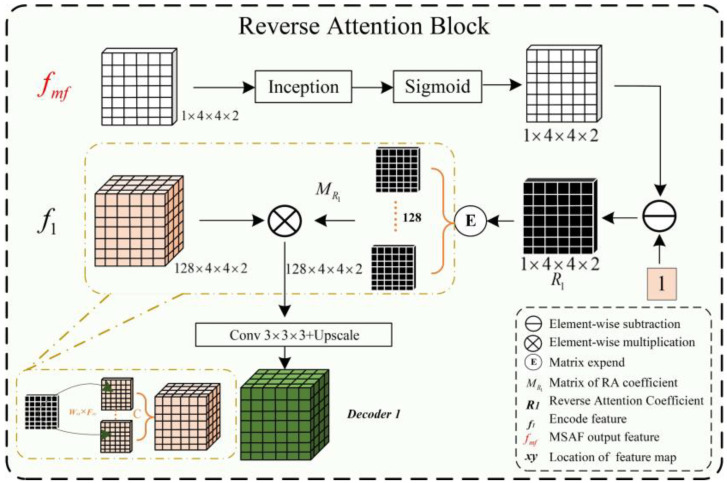
Structural diagram of the Reverse Attention block.

**Figure 5:**
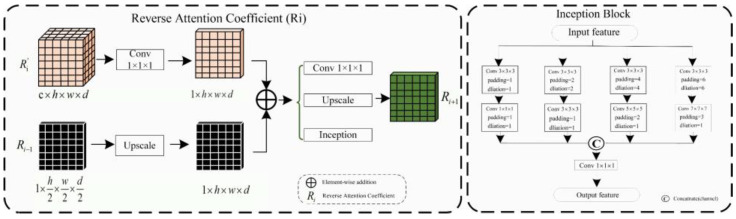
Reverse Attention Coefficient transfer structure and the inception sparse convolution structure.

As shown in Figure 4, the reverse attention module mainly obtains adaptive reverse attention coefficients via feature manipulation. It assigns new weights to input features using these coefficients to enhance the expression ability of edge features, emphasizing the boundary features. Specifically, the multi-scale aggregated feature $f_{mf}\in P^{1\times 4\times 4\times 2}$ is first input into the inception sparse convolution module, which includes multiple dilated convolution structures that can further fuse multi-scale features and enhance feature expression ability. Then, the normalized and inverted features passed through the sigmoid function are used as the reverse attention coefficient $R_{1}\in P^{1\times 4\times 4\times 2}$ to erase foreground features. The reverse attention coefficient $R_{1}$ is extended by channels to obtain $M_{R_{1}}\in P^{128\times 4\times 4\times 2}$, which is pixel-wise multiplied with the input encoded feature $f_{1}\in P^{128\times 4\times 4\times 2}$ to assign new weights to each pixel. Subsequently, the new feature matrix is input into the Conv3 × 3 × 3 convolution and up-sampled to obtain the decoded feature Decoder1. Meanwhile, the left image in Figure 5 describes the process of reverse attention propagation, which $M_{R_{i}}\in P^{c\times h\times w\times d}(R_{i}^{'})$ is restored $R_{i}$ by a 1 × 1 × 1 convolution kernel and $R^{}{}_{i-1}\in P^{1\times \frac{h}{2}\times \frac{w}{2}\times \frac{d}{2}}$ is added to $R_{i}$ after up-sampling. Finally, $R^{}{}_{i+1}\in P^{1\times 2h\times 2w\times 2d}$ is obtained through Conv1 × 1 × 1 convolution, up-sampling, and input into the inception structure. Therefore, the reverse attention mechanism can further enhance feature expression ability and improve segmentation accuracy in segmentation tasks.

(5) $$R_{1}=1-Sigmoid[Inception(f_{mf})]$$

(6) $$Decoder=Conv_{3\times 3\times 3}[Upscale(f_{1}*M_{R_{1}})]$$

(7) $$R_{i}=F[Conv_{3\times 3\times 3}(R_{i}^{'})+Upscale(R_{i-1})]$$

Here *, $f_{mf}$, $R_{i}$, $M_{R_{i}}$, Decoder1, and F represent the functions of matrix multiplication, fused feature, reverse attention coefficient, reverse attention coefficient matrix, decoder feature, convolution, and up-sampling, respectively.

(C) Inception Block

In our reverse attention module, we incorporated the Inception structure as a sparse network to efficiently use computational resources and improve the network’s performance. Since the high-precision multi-scale and edge detail segmentation is crucial in tubular structure segmentation, we fused the Inception structure into the UARAI network architecture to effectively combine multi-scale features. This improved the feature expression without increasing the number of parameters and expanded the network’s receptive field. In the UARAI network structure, the Inception structure is mainly used in the further comprehensive fusion of multi-scale features after multi-scale feature aggregation and the sparse propagation path of reverse attention. As illustrated in Figure 5 (right), the specific structure contains four branches, each consisting of two layers. Each branch layer undergoes processing using convolutions and dilated convolutions with different kernel sizes, followed by spatial and channel-wise fusion of the branch’s results.

(D) Loss function

In this study, tubular structure segmentation suffered from the imbalance between positive and negative samples. The number of foreground pixels belonging to cerebrovascular and airway structures is far less than that of background pixels. Using Dice loss as the loss function can solve this problem and improve segmentation accuracy. Dice loss is a measure of similarity that calculates the similarity between two sets of foreground and background pixels, which has robustness in addressing class imbalance issues. The formula for the Dice loss is as follows:

(8) $$Dsc(gt,pred)=\frac{2\sum_{i=1}^{N}g_{i}p_{i}}{\sum_{i=1}^{N}g_{i}^{2}+\sum_{i=1}^{N}p_{i}^{2}}$$

(9) Dice_loss=1−Dsc(gt,pred)

Here gt, pred, $p_{i}$, and $g_{i}$ respectively represent ground truth, predicted, predicted image pixel, and labeled image pixel.

## 4. Experimental Design

### 4.1. Experimental and Parameter Settings

The experiment is primarily based on MRA and CT images. It aims to validate the effectiveness of our method’s data pre-processing, network model framework (including multi-scale feature fusion, reverse attention, and sparse convolution), and segmentation results’ post-processing. We have conducted a large number of comparative experiments.

All experiments were performed on an A100 GPU with a memory size of 40 G, using CUDA version 11.4 and Python version 3.9. Firstly, 61,864 MRA image patches and 98,852 CT image patches were obtained by combining sliding window sequential cropping combined with random cropping. Secondly, the training parameters were set as follows: the batch size was 100, the epoch was 200, and the Adam optimizer was used for training with an initial learning rate of 0.001. Moreover, dropout = 0.3 was set in the network structure to force the neural network to actively discard some nodes, avoid overfitting deep neural networks, and enhance network generalization. The Early Stop (counters = 50) strategy was adopted during training to prevent overfitting and enhance model robustness and generalization.

### 4.2. Comparative Experiment

To obtain a more objective and reliable tubular structure segmentation model, this study designed three-dimensional comparative experiments based on cerebrovascular and airway datasets, including network dimension-based comparative experiments, patch-cropping method-based comparative experiments, and patch-size-based comparative experiments. These three dimensions are not completely independent but are interrelated, as described below:

(a) Network dimension-based comparative experiments: Based on commonly used medical image segmentation networks, this experiment compared and analyzed the performance of VoxResnet [59], Resnet [60], 3D U-Net [13], Attention U-Net [17], Rattention U-Net [50], CS2-Net [35], ER-Net [36], APA U-Net [39], and the UARAI network proposed in this study. Vessel and airway segmentation are evaluated to thoroughly validate the proposed model’s segmentation effect.
(b) Patch-cropping method-based comparative experiments: In order to verify the influence of different patch acquisition methods on model performance, two comparative experiments were designed in this paper. One method is random patch cropping, and the other combines sequential sliding window cropping and random patch cropping. For random patch cropping, the cropping condition was set as the block threshold greater than 0.01 (as shown in Equation (11)), and a total of 150 patches were cropped for each image. This patch type mainly includes coarse tubular structures with fewer vessels and airways in peripheral areas. The other combination method is to sequentially crop samples with a window size of 64 × 64 × 32 and a step size of 32. Then, 30 samples were randomly cropped from each image, and the threshold was set to 0.001 (no need to set a strict threshold). This strategy can obtain all the feature information of the image quickly and increase sample diversity.
(c) Patch-size-based comparative experiments: Cerebrovascular structures are distributed very sparsely in the brain, and the volume fraction of physiological brain arterial vessels is 1.5%. The voxel resolution of arterial vessels that TOF-MRA can detect can be as low as 0.3% of all voxels in the brain [8]. In addition, cerebrovascular and airway structures are complex, and many tubular structures are of different thicknesses. Samples of different sizes cover different features. Smaller patch sizes contain less context information and focus more on detailed features. In comparison, larger patch sizes contain more global features but have a lower training efficiency and require more dimensionality reduction for obtaining high-level features during down-sampling. Therefore, this experiment designed comparative experiments at different patch sizes of 16 × 16 × 32, 32 × 32 × 32, 64 × 64 × 32, 96 × 96 × 32, and 128 × 128 × 32 to explore the performance differences of the network model under different patch sizes.

### 4.3. Evaluation Metrics

Common semantic segmentation metrics were used in the experiment, including Recall (also known as sensitivity), Precision (also known as positive predictive value or PPV), Dice score, and IoU (intersection over union). These metrics can be used to evaluate the quality of segmentation results. The calculation formulas for each metric are as follows:

(10) $$\begin{array}{l} Recall=\frac{TP}{TP+FN}\\ Precision=\frac{TP}{TP+FP}\\ Dice=\frac{2TP}{2TP+FP+FN}\\ Iou=\frac{gt\cup pred}{gt\cap pred} \end{array}$$

Here TP, FP, TN, FN, gt and pred respectively represent true positive, false positive, true negative, false negative, and ground truth predicted.

## 5. Results

This study conducted several comparative experiments on cerebrovascular MRA and lung CT image datasets to verify the effectiveness of our proposed method. To ensure fairness, we randomly partitioned the training, validation, and testing data in the same hardware environment and used consistent evaluation metrics and post-processing methods for comparative analysis. Precision (Pre), Recall (Re), Dice score (Di), and IoU were used as the evaluation metrics for segmentation effectiveness.

### 5.1. Cerebrovascular and Airway Segmentation Results

**Figure 6:**
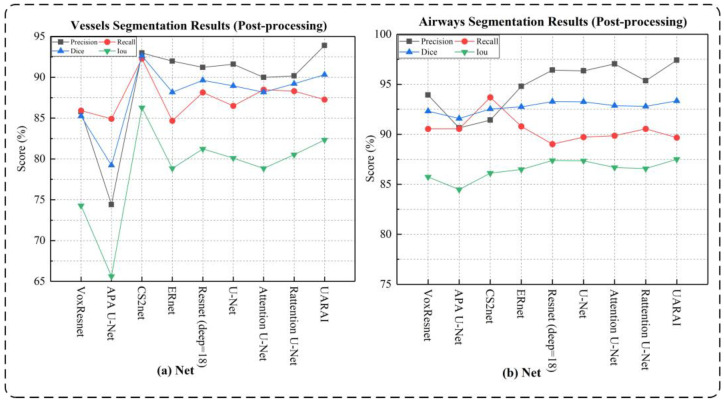
Comparison of cerebrovascular and airway segmentation performance under different networks in the size of 64 × 64 × 32, where (**a**) is the cerebrovascular results, and (**b**) is the airway results.

**Figure 7:**
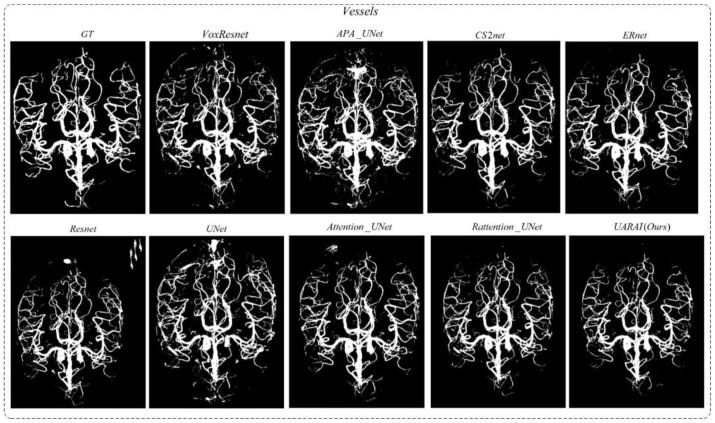
Cerebrovascular segmentation results in different networks (without post-processing).

**Figure 8:**
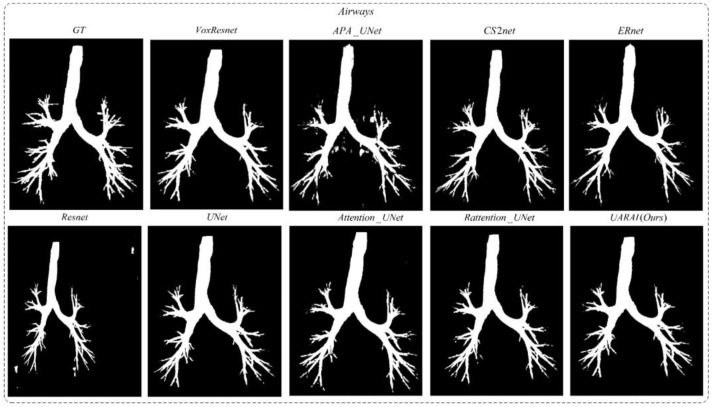
Airway segmentation results in different networks (without post-processing).

Comparison experiment of network: Through comparison with other networks using the same post-processing method, we found that in the task of cerebral vascular segmentation (as shown in Table 2 and Figure 6). The network segmentation comparison results without post-processing are shown in Figure 7 and Figure 8. Our proposed method outperformed U-Net by 2.29%, 1.36%, and 2.23% in Precision, Dice, and IoU, respectively. It also achieved higher performance than VoxResnet, with 8.09%, 5.07%, and 8.05% improvements in Precision, Dice and IoU, respectively, as well as Resnet, with 2.77%, 0.68%, and 1.11% improvements; Attention U-Net, with 3.91%, 2.14%, and 3.49% improvements; Rattention U-Net, with 3.72%, 1.10%, and 1.81% improvements; CS2-Net, with 0.74%, 2.41%, and 3.91% improvements; ER-Net, with 1.91%, 2.14%, and 3.49% improvements; and APA U-Net, by significant margins of 19.48%, 11.09%, and 16.71% in Precision, Dice, and IoU, respectively. 

Similarly, in the task of airway segmentation, our proposed method achieved the best performance and outperformed U-Net, with 1.07%, 0.09%, and 0.16% improvements in Precision, Dice, and IoU, respectively, as well as VoxResnet, with 3.49%, 1.02%, and 1.77% improvements; Resnet, with 0.99%, 0.07%, and 0.12% improvements; Attention U-Net, with 0.37%, 0.47%, and 0.82% improvements; Rattention U-Net, with 2.06%, 0.54%, and 0.94% improvements; CS2-Net, with 5.99%, 0.80%, and 1.39% improvements; ER-Net, with 2.62%, 0.59%, and 1.02% improvements; and APA U-Net, with 6.76%, 1.76%, and 3.03% improvements. In summary, our proposed UARAI model achieved superior segmentation performance in terms of Precision, Dice, and IoU compared to other models, particularly in the task of cerebral vascular segmentation. It also exhibited some improvement in the task of airway segmentation.

**Figure 9:**
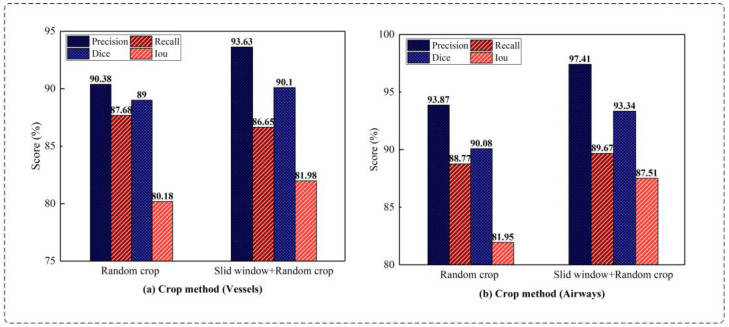
Comparing the experimental results of different cutting methods. Among them, (**a**) shows the comparison results of ‘random cropping’ and ‘sliding window + random cropping’ for cerebral vessels; (**b**) shows the comparison results of ‘random cropping’ and ‘sliding window + random cropping’ for airways (without post-processing).

Comparison of cropping methods: This experiment uses the UARAI structure with a training sample size of 64 × 64 × 32. Various cutting methods are compared, and the segmentation results are presented in Figure 9. Among these methods, combining the ‘sliding window sequence + random’ cutting method yields the highest Precision, Dice, and IoU scores on the cerebrovascular segmentation dataset. Specifically, the Precision, Dice, and IoU values are 93.63%, 90.10%, and 81.98%, respectively. Compared to the ‘random’ cutting method, there is an improvement of 3.25%, 1.10%, and 1.80% in Precision, Dice, and IoU scores, respectively, while the Recall value slightly decreases by 1.03%. Among the airway dataset, the segmentation results obtained through the combination of the ‘sliding window sequence + random’ cutting method yield the highest Precision, Recall, Dice, and IoU scores, which are 97.41%, 89.67%, 93.34%, and 87.51% respectively. These scores reflect improvements of 3.54%, 0.9%, 3.26%, and 5.56% compared to the results obtained using the ‘random’ cutting method.

**Figure 10:**
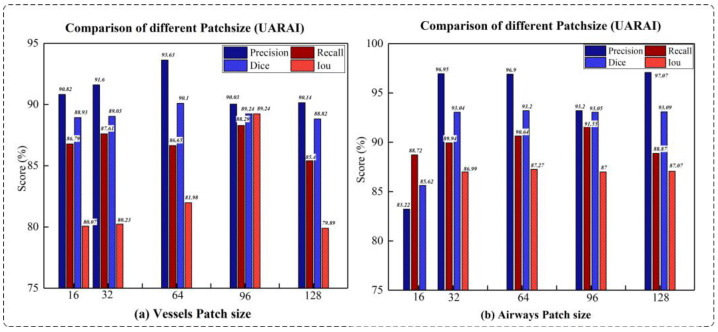
Based on comparing different patch-size segmentation results under UARAI, (**a**) is the cerebrovascular results, and (**b**) is the airway results.

Comparison experiment of patch size: We compared the segmentation results of the model on different cross-sectional sizes of cerebrovascular and airway samples, as shown in Table 3 and Table 4. Regarding segmentation performance, the training sample size of 64 × 64 × 32 demonstrates the best results, disregarding the z-axis dimension. Specifically, in the cerebrovascular dataset, the size of 64 × 64 × 32 yields the highest Precision, Recall, Dice, and IoU scores, which are 93.63%, 89.29%, 90.10%, and 81.98%, respectively. For the airway dataset, the size of 64 × 64 × 32 achieves the best Dice and IoU scores of 93.20% and 87.27%, respectively. However, the Precision reaches its peak at the size of 128 × 128 × 32, standing at 97.07%, while the highest Recall is attained at the size of 96 × 96 × 32, amounting to 91.55%. Figure 10 visually illustrates the segmentation outcomes of the cerebrovascular and airway datasets obtained through the UARAI network, considering different patch sizes employed in this experiment. These results solidify the superiority of the training sample size of 64 × 64 × 32 for the cerebrovascular and airway datasets used in this study.

### 5.2. Ablation Studies

**Figure 11:**
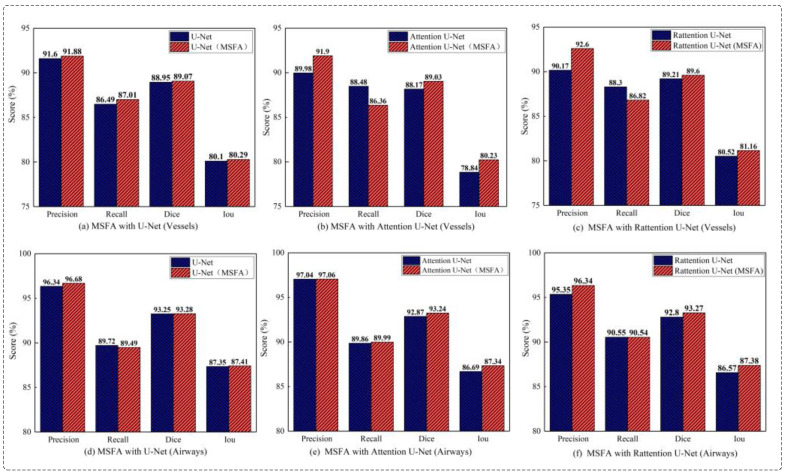
The ablation experiment on MSFA yielded significant results. In cerebrovascular segmentation, (**a**–**c**) correspond to the comparative outcomes of ‘MSFA’ across U-Net, Attention U-Net, and Rattention U-Net, respectively. Similarly, in airway segmentation, (**d**–**f**) represent the comparison results of ‘MSFA’ within U-Net, Attention U-Net, and Rattention U-Net, respectively.

To assess the efficacy of each module, this study conducted ablation experiments on the multi-scale feature aggregation module (MSFA) and the reverse attention sparse convolution module (Ra + Icp) within the cerebrovascular and airway segmentation models. The standard model, built upon the 3D U-Net baseline network framework, encompassed the modules’ Baseline + MSFA + Ra + Icp’. The ablation experiments were carried out as follows:

Ablation studies of MSFA: This study compared the effectiveness of the MSFA module in Attention and Reverse Attention network structures and verified the consistency of multi-scale feature aggregation in improving segmentation accuracy for different tubular objects. In the ablation experiments, the MSFA module was integrated into ‘Baseline’, ‘Baseline + Attention’, ‘Baseline + Rattention’, and the standard model. Table 5 presents the segmentation results for various models in both cerebrovascular and airway segmentation. In Figure 11, the models are depicted in red and blue, representing those with and without MSFA (multi-scale feature aggregation). The result demonstrated that the MSFA module could enhance the network’s representation ability for different scales and improve segmentation accuracy for cerebrovascular and airway segmentation tasks.

**Figure 12:**
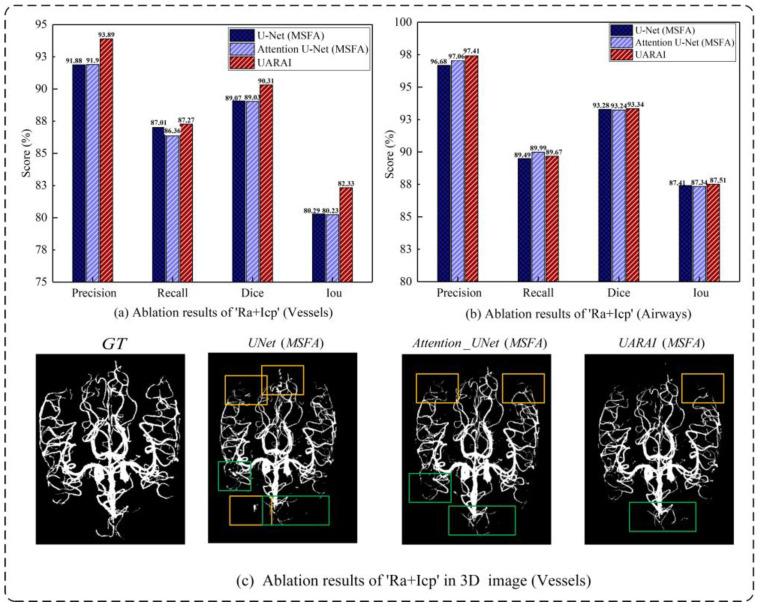
The ablation experiment on ‘Ra + Icp’ yielded insightful results. In cerebrovascular and airway segmentation, (**a**,**b**) showcase the comparative outcomes of ‘Ra + Icp’. Furthermore, (**c**) illustrates the visualization of the cerebrovascular segmentation results. The models used for evaluation include U-Net (MSFA) as the ‘Baseline + MSFA’ model, Attention U-Net (MSFA) as the ‘Baseline + MSFA + Attention’ model, and UARAI as the ‘Baseline + MSFA + Ra + Icp’ model. False positive and false negative areas are highlighted by yellow and green boxes, respectively.

Ablation studies of ‘Ra + Icp’: Utilizing multi-scale feature aggregation, this study comprehensively evaluated and compared the efficacy of the Ra + Icp module in the ‘Baseline + MSFA’, ‘Baseline + MSFA + Attention’ and ‘Baseline + MSFA + Ra + icp’ models. The findings in Table 6 and Figure 12 indicate that the ‘Ra + Icp’ module significantly enhances cerebrovascular segmentation. The model demonstrates notable improvements in Precision, Recall, Dice, and IoU indices, exhibiting a respective increase of 2.01%, 0.26%, 1.24%, and 2.04% over the ‘Baseline + MSFA’ model. Moreover, compared with ‘Baseline + MSFA + Attention’, the model achieves a boost of 1.99% in Precision, 0.9% in Recall, 1.28% in Dice, and 2.1% in IoU indices. Conversely, for airway segmentation, the impact of the ‘Ra + Icp’ module is slightly less pronounced. The model shows improvements of 0.73% in Precision, 0.18% in the Recall, 0.06% in Dice, and 0.1% in IoU indices when compared with ‘Baseline + MSFA’ while achieving enhancements of 0.35% in Precision, 0.1% in Dice, and 0.17% in IoU indices compared with ‘Baseline + MSFA + Attention’. However, it should be noted that the Recall index experiences a slight decrease in this context.

**Figure 13:**
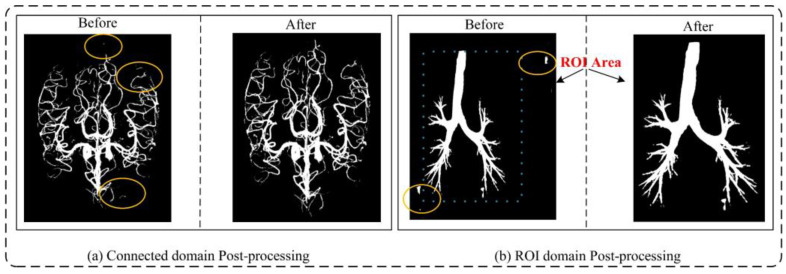
A comprehensive comparison between pre- and post-processing results reveals intriguing insights. In particular, (**a**) showcases the refined cerebrovascular prediction outcomes achieved through connected domain processing, while (**b**) demonstrates the improved airway prediction results obtained by leveraging the region of interest (ROI) encompassing the lung parenchyma, including the airways. This meticulous analysis highlights the significant impact of post-processing techniques in enhancing the accuracy and reliability of the predictions. False positive is highlighted by yellow circle.

Ablation studies of Post-processing: Post-processing techniques play a crucial role in enhancing the outcomes of medical image segmentation. This study employed two key post-processing strategies to refine the results. The first strategy involved applying adaptive filtering to address false positive regions based on the original image’s region of interest (ROI). This approach effectively mitigated isolated pixel areas that tend to emerge within cerebrovascular and airway regions. The second strategy focused on removing isolated pixel points by considering the maximum connected domain. By implementing these strategies, the segmentation results exhibited improved accuracy by effectively handling false positive areas outside the brain tissue and lung parenchyma, as depicted in Figure 13. To quantify the impact of post-processing, Table 2 showcases the results obtained by applying identical post-processing techniques across different network training sessions, utilizing a patch size of 64 × 64 × 32. Furthermore, Table 2 highlights significant advancements in the associated measurements compared with Table 4 and Table 5 with Resnet displaying outstanding performance.

## 6. Discussion

Cerebrovascular and airway segmentation has always been a significant clinical concern. To address the challenge of low segmentation accuracy due to the complexity of the cerebrovascular and airway structures and the difficulty in extracting features from end and edge regions, we suggest a multi-scale feature aggregation reverse attention sparse convolution network architecture that can enhance feature extraction for tubular structures with varying thicknesses and complex shapes. As a result, this method can enhance the expression ability of edge features, leading to high-precision segmentation of cerebrovascular and airway structures. The proposed network structure achieved Dice and IoU scores of 90.31% and 82.33%, respectively, in cerebrovascular segmentation. In airway segmentation, the Dice and IoU scores were 93.34% and 87.51%, respectively. The results suggest that the approach surpasses the commonly used segmentation networks. Furthermore, the findings indicate that the proposed method can accurately segment tubular structures, which is crucial in clinical diagnosis, preoperative planning, and prognosis analysis.

The primary objective of this study is to tackle the challenge of accurate segmentation of tubular structures, despite the limited availability of medical imaging data. To overcome this challenge, we propose a novel segmentation strategy that combines a sliding window sequence with random cropping, enabling us to generate a diverse and extensive range of training samples. By utilizing a patch size of 16 × 16 × 32, sliding window steps of 16, and random cropping of 30, we successfully obtained a remarkable 223,896 training samples. Similarly, with a patch size of 64 × 64 × 32 and sliding window steps of 32, we acquired 46,056 training samples. Moreover, leveraging a patch size of 128, sliding window steps of 64, and random cropping of 30 resulted in 9880 training samples. These findings unequivocally demonstrate that our proposed method generates a significantly larger sample pool than conventional random cropping techniques.

We integrated multiple image-enhancement techniques into the training process to further enrich the training samples and enhance the model’s generality. These techniques played a crucial role in augmenting the training samples and boosting their representativeness. Experimental outcomes based on different patch sizes indicated that the optimal segmentation performance was achieved at a resolution of 64 × 64, irrespective of the layer thickness along the z-axis.

We conducted a comparative analysis of two patch extraction techniques: random cropping and random cropping combined with sliding window sequential cropping. In the case of random cropping, patches were extracted by determining a threshold based on the ratio of label pixels to the total number of pixels within each patch (as shown in Equation (11)). The choice of the threshold value directly influenced the accuracy of the segmentation. We found that extremely low or high values had a negative impact on the experimental results. If the threshold value was set too low, the resulting patches mainly consisted of background regions, lacking sufficient image feature information for effective training. On the other hand, an excessively high threshold value led to longer cropping times, reducing the efficiency of training. Additionally, since the background area is a significant component of the segmentation task, the random cropping approach often overlooked the background area, resulting in inconsistencies between the training patches and the actual image features. As a result, this approach led to decreased prediction accuracy.

To address these challenges, we adopted a sliding window sequential cropping approach and a non-strict threshold random cropping strategy when extracting patches for cerebrovascular and airway segmentation. Initially, the sliding window technique was employed to extract patches, ensuring the comprehensive inclusion of image feature information pertaining to the tubular structures of interest. Additionally, we incorporated a limited amount of random cropping to introduce diversity among the samples. This combined approach effectively captured all relevant image features. By adopting a more lenient threshold in random cropping, we successfully mitigated the issues mentioned above, leading to improved segmentation accuracy and preserving the necessary diversity in the training data.

(11) $$Threshold=\frac{\sum_{i=0,j=0,k=0}^{h,w,d}V_{patch}(i,j,k)}{V_{crop(h,w,d)}}$$

Here, Threshold, $V_{patch}(i,j,k)$, $V_{patch}(i,j,k)$, and $V_{crop}$ respectively represent the threshold value set for random patch cropping, the corresponding label pixel value of each pixel in the patch, and the size of the patch.

Figure 9 demonstrates that the fusion of sliding window sequential cropping and random cropping techniques yielded exceptional outcomes in cerebrovascular segmentation. The combined cropping strategy showcased notable improvements in various evaluation metrics compared with the sole utilization of random cropping. Specifically, the Dice score saw a commendable enhancement of 1.1%, Precision witnessed a substantial boost of 3.25%, and IoU experienced a significant increase of 1.8%. However, it is worth mentioning that the Recall exhibited a marginal decrease of 1.03% in this case.

In airway segmentation, employing the model trained to integrate sliding window sequential cropping and random cropping led to impressive results. Notably, there were remarkable improvements across multiple performance measures. The Dice score witnessed a substantial surge of 3.26%, Precision increased by an impressive 3.54%, IoU experienced a noteworthy boost of 5.56%, and Recall demonstrated a favorable increment of 0.9%, compared with the performance achieved solely through random cropping. These findings strongly indicate the efficacy and superiority of the combined cropping strategy in enhancing the segmentation accuracy for both cerebrovascular and airway datasets.

Our experimental findings shed light on the significant impact of patch size selection on the sensitivity of cerebrovascular and airway segmentation. Previous research [24] has emphasized that a smaller cropping size prompts the network to focus predominantly on local features. In comparison, a larger cropping size enables the network to capture more global features, albeit at the potential cost of requiring additional max-pooling layers. In our study, we conducted extensive comparative experiments on brain vasculature and airway datasets to determine the optimal cropping size for these specific domains.

**Figure 14:**
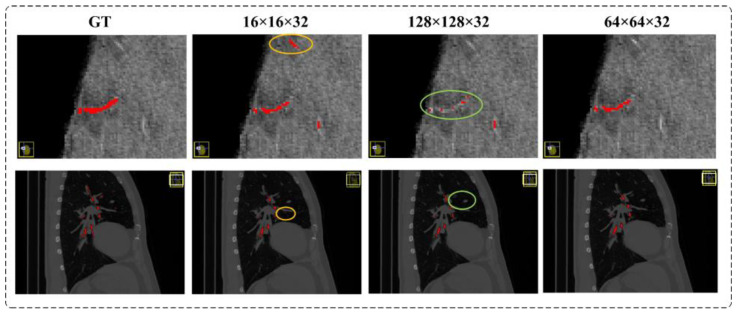
This sequence provides a comprehensive visual representation of the performance of the different patch sizes. The evaluation of training results, relative to the ground truth label (GT), is depicted in a comparative manner from left to right, highlighting models trained with patch sizes of 16 × 16 × 32, 128 × 128 × 32, and 64 × 64 × 32, respectively. False positive and false negative areas are highlighted by yellow and green circles, respectively.

The results unequivocally establish that a model with a patch size of 64 × 64 × 32 achieves superior segmentation accuracy by adeptly capturing global and intricate features in a well-balanced manner. This conclusion is substantiated by the compelling evidence presented in Table 3 and Table 4, which consistently highlight enhanced segmentation performance when utilizing the 64 × 64 × 32 size. Moreover, Figure 14 visually illustrates the segmentation outcomes achieved by models trained with different patch sizes. In Figure 14, the yellow circle represents false positives, while the green circle signifies false negatives. Our findings underscore that a cropping size of 64 × 64 × 32 yields the most favorable segmentation results, characterized by minimal false positives and false negatives.

It is essential to note that using small patches may lead to a higher incidence of false positives, primarily due to the network’s limited ability to comprehend contextual cues from these diminutive patches. Consequently, neighboring background regions might be classified as tubular structures erroneously, thereby contributing to false positive predictions. Conversely, larger patches encompass a greater degree of background interference, impeding the network’s capacity to accurately discern finer details of the tubular structures. Consequently, there is a propensity for misidentifying cerebrovascular and airway regions as background, leading to elevated false negative rates. Thus, our findings underscore the crucial role played by the selection of an appropriate cropping size, with the 64 × 64 × 32 dimensions proving to be optimal for achieving accurate and reliable segmentation outcomes.

**Figure 15:**
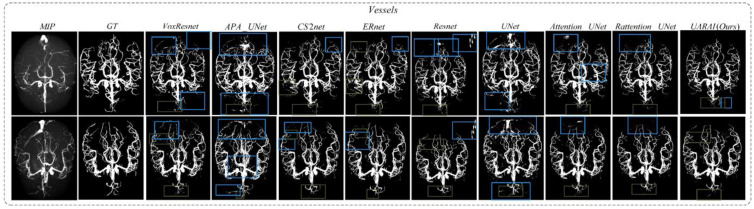
The results obtained from various models in cerebrovascular segmentation revealed distinct patterns. Blue and green boxes depict false positive and false negative areas, respectively, providing a visual representation of the discrepancies among the models. False positive and false negative areas, particularly at the edges, are highlighted by blue and dark green boxes, respectively.

**Figure 16:**
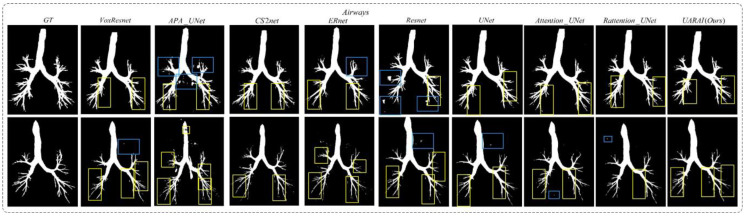
The outcomes of different models in airway segmentation illustrate noteworthy variations. False positive and false negative areas, particularly at the edges, are highlighted by blue and yellow boxes, respectively, offering insight into the performance disparities among the models. False positive and false negative areas, particularly at the edges, are highlighted by blue and yellow boxes, respectively.

In addition, to comprehensively validate the effectiveness of the proposed method in this paper, the proposed network was compared with existing segmentation methods. As shown in Figure 15 and Figure 16, there were differences in the false positive and false negative cases among different networks. In Figure 15, we present two sets of cerebrovascular image segmentation results. The first column shows the maximum intensity projection (MIP) image of brain vasculature, which displays the distribution of blood vessels in the brain. The second column shows the ground truth labels and the subsequent columns show the segmentation results of various networks. Specifically, the U-Net model performs well in medical image segmentation and has good overall segmentation results but performs slightly worse in edge segmentation, small blood vessel segmentation, and airway segmentation. Although the U-Net model performs well in segmenting the primary vascular branches and airways, its ability to segment tubular structures near the edges is suboptimal.

The U-Net and APA U-Net model exhibited limited discrimination ability when segmenting the vascular region at the arteriovenous junction during cerebral vascular segmentation. This limitation led to a higher occurrence of false positives in the results. On the other hand, the VoxResnet model showcased superior segmentation outcomes compared with the U-Net model, effectively reducing the occurrence of false positives. This improvement can be attributed to the presence of residual connections within its architecture, which mitigated the lack of shallow feature information and enhanced the segmentation accuracy. Additionally, increasing the depth of the Resnet model with residual connectivity further reduced the incidence of false positives in the predicted outcomes. However, a larger false positive region emerged outside the non-brain and non-airway regions, possibly due to the increased complexity of deeper network layers and the imbalanced ratio of positive and negative samples.

In Figure 15, two sets of three-dimensional vessel segmentation results are presented. In the CS2-Net model, the network addresses the weak segmentation ability of U-Net and APA U-Net at the intersection of arteries and veins by utilizing both spatial and channel attention, significantly reducing false positive cases and improving segmentation accuracy. In ER-Net, the use of reverse attention enhances the edge feature module, further improving the segmentation ability for edge blood vessels and reducing false positive cases, but there still exist some false negative cases. Examining the segmentation results of the Attention U-Net and Rattention U-Net models, noticeable enhancements were observed in the segmentation accuracy of edge details, accompanied by a significant reduction in the false positive rate. In the case of the UARAI model segmentation results, a substantial decrease in the number of isolated false positive areas was evident. Moreover, the segmentation of small blood vessels became more delicate and accurate, and the segmented blood vessels exhibited improved continuity aligned with the anatomical structure characteristics. However, a few false negative cases persisted, which could be attributed to the challenge of differentiating arterial and venous image features that share similarities.

Moving to Figure 16, two sets of three-dimensional airway segmentation results are presented. Predominantly, the airway segmentation outcomes exhibit more false negatives and fewer false positives. Overall, all networks’ main airway segmentation results demonstrate improved accuracy, although the segmentation of small airways falls short of ideal performance. In the APA U-Net and ResNet networks, there are many false positive regions outside the airway, which greatly affect segmentation performance. After post-processing, the accuracy is greatly improved. The false positive cases in the segmentation results of the U-Net and VoxResNet models are greatly improved, but the performance of edge segmentation still needs to be improved. The CS2-Net, ER-Net, Attention U-Net, and Rattention U-Net models introduced different attention mechanisms, which improved overall performance compared with U-Net. Particularly, in the ER-Net and Rattention U-Net models, the edge segmentation accuracy is significantly improved, further confirming the reusability of reverse attention in complex tube-like structures and edge detail segmentation. Notably, the UARAI model demonstrated exceptional performance in edge detail segmentation and the segmentation of small airways, as depicted in the yellow box area. Additionally, the false positive rate in the segmentation results was notably low, as indicated by the blue box area, resulting in highly accurate segmentation outcomes.

Under the UARAI framework, we conducted comparative experiments on diverse network models. The results indicate a noteworthy advancement in Precision, Dice, and IoU scores; however, we observed a minor decline in Recall as compared to other networks. As previously mentioned, an improvement in Precision indicates more accurate true-positive predictions or fewer false positives, with the model being more focused on predicting positive samples and making stricter judgments, thereby reducing misjudgments. Dice and IoU scores mainly focus on the overlapping area between the model’s prediction results and the ground truth labels. Recall and Precision differ because Recall is more concerned about false-negative areas, with slightly lower values indicating that the model missed several positive samples and suffered from slight under-segmentation.

Low image resolution and large pixel spacing in cerebrovascular and airway datasets may create peripheral marker discontinuity. This leads the model to ignore positive areas that lack markers and treat them as background. This, in turn, affects the Recall value and the segmentation accuracy of tubular structures. Future work needs to address these challenges in achieving higher accuracy segmentation of tubular structures. To that end, we will focus on conducting semi-supervised methods that will primarily tackle issues relating to image quality and labeling limitations. For instance, we can employ self-training by utilizing semi-supervised learning to generate highly confident pseudo-labels repeatedly. Alternatively, we can use perturbation-consistent semi-supervised training methods to solve such issues and improve segmentation accuracy.

## 7. Conclusions

This research paper introduces a novel approach for accurately segmenting tubular structures such as cerebrovascular and airway structures. To address the challenges posed by complex tubular objects, we employed a combination of sliding window sequential cropping and random cropping strategies to increase the number of training samples and leverage the available image features effectively. Additionally, we proposed a unique U-Net-based framework that incorporates multi-scale feature aggregation, reverse attention, and sparse convolution. A comprehensive experimental analysis was conducted to evaluate the efficacy of different components, including data pre-processing, model framework, and post-processing techniques.

The introduction of multi-scale feature aggregation enables the network to learn and adapt to different shapes and thicknesses of tubular structures at varying scales, enhancing the overall feature learning process. Incorporating reverse attention allows the model to dynamically emphasize edge features, improving the extraction of positive samples and edge details. Furthermore, integrating Inception sparse convolution enhances the network’s receptive field and feature representation without significantly increasing model complexity.

Extensive experiments were conducted on cerebrovascular and airway datasets, demonstrating promising results. The proposed UARAI model achieved impressive Dice and IoU scores of 90.31% and 82.35% (cerebrovascular) and 93.34% and 87.60% (airways), respectively. Comparative analysis with existing advanced methods showcased the superior segmentation accuracy of our proposed model. Consequently, our proposed method can be regarded as an effective approach for tubular structure segmentation, offering advancements in accuracy and paving the way for improved medical image analysis and diagnosis. 

## Figures and Tables

**Table 1:** Cerebrovascular dataset and airway dataset.

Dataset	Image Size	Patch Size	Number of Training	Number of Validation	Number of Test	Number of Training Patches
Cerebrovascular (MIDAS)	448 × 448 × 128	64 × 64 × 32	76	11	22	46,056
Airway (GMU)	512 × 512 × 320	64 × 64 × 32	265	38	77	98,852

**Table 2:** Segmentation results of cerebrovascular and airway structures by different networks (including post-processing); the evaluation index is the average value and variance of the prediction results of the test set; MSFA means multi-scale feature aggregation and the red bold is the optimal result (post-processing).

	Vessels Dataset				Airways Dataset			
Network	Pre (%)	Re (%)	Di (%)	IoU (%)	Pre (%)	Re (%)	Di (%)	IoU (%)
VoxResnet [59]	85.80 ± 1.92	85.92 ± 1.78	85.24 ± 1.11	74.28 ± 1.64	93.92 ± 2.13	90.54 ± 3.12	92.32 ± 2.03	85.74 ± 2.74
APA U-Net [39]	74.41 ± 3.86	84.91 ± 1.63	79.22 ± 1.67	65.62 ± 2.26	90.65 ± 7.71	90.55 ± 7.98	91.58 ± 6.87	84.48 ± 8.57
CS2-Net [35]	93.15 ± 1.25	83.23 ± 1.20	87.90 ± 0.67	78.42 ± 1.06	91.42 ± 1.95	**93.70 ± 2.01**	92.54 ± 2.00	86.12 ± 2.79
ER-Net [36]	91.98 ± 1.44	84.67 ± 1.22	88.17 ± 0.57	78.84 ± 1.44	94.79 ± 2.48	90.80 ± 5.14	92.75 ± 3.53	86.49 ± 5.46
Resnet (deep = 18) [60]	91.21 ± 1.18	88.14 ± 1.66	89.63 ± 0.81	81.22 ± 1.33	96.42 ± 5.79	89.02 ± 3.27	93.27 ± 2.54	87.39 ± 3.22
U-Net [13]	91.60 ± 1.92	86.49 ± 1.03	88.95 ± 0.89	80.10 ± 1.44	96.34 ± 0.65	89.72 ± 3.15	93.25 ± 1.84	87.35 ± 3.15
Attention U-Net [17]	89.98 ± 1.35	**88.48 ± 1.52**	88.17 ± 0.45	78.84 ± 1.05	97.04 ± 0.61	89.86 ± 3.72	92.87 ± 2.25	86.69 ± 3.68
Rattention U-Net [50]	90.17 ± 1.39	88.30 ± 1.23	89.21 ± 0.60	80.52 ± 0.99	95.35 ± 5.21	90.55 ± 2.97	92.80 ± 3.51	86.57 ± 5.50
UARAI (Ours)	**93.89 ± 1.22**	87.27 ± 2.15	**90.31 ± 0.82**	**82.33 ± 1.37**	**97.41 ± 0.56**	89.67 ± 3.37	**93.34 ± 1.98**	**87.51 ± 3.34**

**Table 3:** In comparing the network segmentation results of the cerebrovascular dataset under different patch sizes (without post-processing), the red bold is the best result.

Patch Size	16 × 16 × 32				32 × 32 × 32			
Network	Pre (%)	Re (%)	Di (%)	IoU (%)	Pre (%)	Re (%)	Di (%)	IoU (%)
VoxResnet [59]	83.75 ± 1.95	85.76 ± 1.40	84.73 ± 1.24	73.51 ± 1.84	83.75 ± 1.95	85.76 ± 1.40	84.73 ± 1.24	73.51 ± 1.84
Resnet (deep = 18) [60]	82.10 ± 1.78	79.40 ± 1.25	80.20 ± 1.96	66.94 ± 2.01	83.40 ± 1.09	80.00 ± 1.56	81.70 ± 1.21	69.06 ± 1.43
U-Net [13]	86.33 ± 1.54	87.17 ± 0.79	86.74 ± 0.72	76.58 ± 1.11	72.27 ± 1.74	79.64 ± 1.05	75.75 ± 0.56	60.97 ± 0.72
Attention U-Net [17]	83.79 ± 2.22	85.85 ± 1.23	84.78 ± 0.82	73.58 ± 1.23	87.39 ± 1.83	84.89 ± 1.55	84.92 ± 0.66	73.79 ± 1.07
Attention U-Net (MSFA)	88.14 ± 1.75	85.86 ± 1.10	85.96 ± 0.77	75.38 ± 1.22	87.98 ± 1.87	85.95 ± 1.64	85.88 ± 0.64	75.25 ± 1.12
Rattention U-Net [50]	86.41 ± 1.00	86.61 ± 0.65	87.90 ± 0.52	78.41 ± 0.51	88.01 ± 2.97	86.40 ± 1.81	88.02 ± 0.93	78.60 ± 1.36
Rattention U-Net (MSFA)	86.47 ± 2.43	**87.71 ± 1.22**	87.97 ± 0.82	78.52 ± 1.22	89.77 ± 1.59	**88.04 ± 1.76**	88.17 ± 0.73	78.84 ± 1.18
UARAI (Ours)	**90.82 ± 1.88**	86.79 ± 0.94	**88.93 ± 1.11**	**80.07 ± 1.74**	**91.60 ± 1.33**	87.61 ± 0.95	**89.03 ± 0.80**	**80.23 ± 1.31**
**Patch Size**	**64 × 64 × 32**				**96 × 96 × 32**			
**Network**	**Pre (%)**	**Re (%)**	**Di (%)**	**IoU (%)**	**Pre (%)**	**Re (%)**	**Di (%)**	**IoU (%)**
VoxResnet [59]	83.75 ± 1.95	85.76 ± 1.40	84.73 ± 1.24	73.51 ± 1.84	83.75 ± 1.95	85.76 ± 1.40	84.73 ± 1.24	73.51 ± 1.84
Resnet (deep = 18) [60]	82.10 ± 1.78	79.40 ± 1.25	80.20 ± 1.96	66.94 ± 2.01	83.40 ± 1.09	80.00 ± 1.56	81.70 ± 1.21	69.06 ± 1.43
U-Net [13]	86.33 ± 1.54	87.17 ± 0.79	86.74 ± 0.72	76.58 ± 1.11	72.27 ± 1.74	79.64 ± 1.05	75.75 ± 0.56	60.97 ± 0.72
Attention U-Net [17]	83.79 ± 2.22	85.85 ± 1.23	84.78 ± 0.82	73.58 ± 1.23	87.39 ± 1.83	84.89 ± 1.55	84.92 ± 0.66	73.79 ± 1.07
Attention U-Net (MSFA)	88.14 ± 1.75	85.86 ± 1.10	85.96 ± 0.77	75.38 ± 1.22	87.98 ± 1.87	85.95 ± 1.64	85.88 ± 0.64	75.25 ± 1.12
Rattention U-Net [50]	86.41 ± 1.00	86.61 ± 0.65	87.90 ± 0.52	78.41 ± 0.51	88.01 ± 2.97	86.40 ± 1.81	88.02 ± 0.93	78.60 ± 1.36
Rattention U-Net (MSFA)	86.47 ± 2.43	**87.71 ± 1.22**	87.97 ± 0.82	78.52 ± 1.22	89.77 ± 1.59	**88.04 ± 1.76**	88.17 ± 0.73	78.84 ± 1.18
UARAI (Ours)	**90.82 ± 1.88**	86.79 ± 0.94	**88.93 ± 1.11**	**80.07 ± 1.74**	**91.60 ± 1.33**	87.61 ± 0.95	**89.03 ± 0.80**	**80.23 ± 1.31**
**Patch Size**	**128 × 128 × 32**							
**Network**	**Pre (%)**	**Re (%)**	**Di (%)**	**IoU (%)**				
VoxResnet [59]	83.75 ± 1.95	85.76 ± 1.40	84.73 ± 1.24	73.51 ± 1.84				
Resnet (deep = 18) [60]	83.10 ± 1.52	74.00 ± 1.12	80.90 ± 0.88	67.93 ± 0.81				
U-Net [13]	79.50 ± 3.60	83.25 ± 3.01	81.24 ± 2.11	68.41 ± 3.02				
Attention U-Net [17]	76.70 ± 2.01	86.42 ± 2.69	81.21 ± 1.15	68.36 ± 1.69				
Attention U-Net (MSFA)	80.45 ± 1.42	86.56 ± 1.23	83.45 ± 0.56	71.60 ± 1.17				
Rattention U-Net [50]	89.20 ± 1.30	85.94 ± 1.70	88.93 ± 0.77	80.07 ± 1.27				
Rattention U-Net (MSFA)	90.02 ± 1.16	**86.79 ± 0.97**	88.82 ± 0.73	79.89 ± 1.19				
UARAI (Ours)	**90.14 ± 1.36**	85.40 ± 1.39	**88.82 ± 0.61**	**79.89 ± 1.00**				

**Table 4:** In comparing the network segmentation results of the airway dataset under different patch-sizes (without post-processing), the red bold is the best result.

Patch Size	16 × 16 × 32				32 × 32 × 32			
Network	Pre (%)	Re (%)	Di (%)	IoU (%)	Pre (%)	Re (%)	Di (%)	IoU (%)
VoxResnet [59]	**93.89 ± 2.03**	**90.65 ± 2.51**	**92.21 ± 1.73**	**85.54 ± 2.99**	93.89 ± 2.03	90.65 ± 2.51	92.21 ± 1.73	85.54 ± 2.99
Resnet (deep = 18) [60]	75.90 ± 6.25	80.25 ± 5.01	79.16 ± 4.98	65.51 ± 6.98	77.54 ± 5.92	85.36 ± 5.21	82.31 ± 5.37	69.94 ± 6.02
U-Net [13]	81.20 ± 7.52	87.17 ± 5.45	83.83 ± 5.10	72.16 ± 7.42	93.81 ± 1.94	89.00 ± 3.13	90.12 ± 2.01	82.02 ± 3.44
Attention U-Net [17]	81.39 ± 7.97	85.88 ± 5.54	80.90 ± 5.46	67.93 ± 7.55	94.44 ± 5.28	90.77 ± 2.65	92.50 ± 3.47	86.05 ± 5.50
Attention U-Net (MSFA)	81.40 ± 6.88	86.23 ± 5.39	84.83 ± 4.33	73.66 ± 6.51	96.86 ± 1.49	89.78 ± 3.14	92.86 ± 1.93	86.67 ± 3.31
Rattention U-Net [50]	54.88 ± 15.99	86.92 ± 5.81	66.37 ± 10.59	49.67 ± 12.78	95.28 ± 3.63	**90.08 ± 2.75**	92.55 ± 2.34	86.13 ± 3.97
Rattention U-Net (MSFA)	62.79 ± 12.16	85.81 ± 4.80	71.08 ± 9.17	55.13 ± 10.28	96.75 ± 1.36	89.77 ± 3.33	93.01 ± 1.97	86.93 ± 3.35
UARAI (Ours)	83.22 ± 7.44	88.72 ± 4.61	85.62 ± 4.54	74.86 ± 6.82	**96.95 ± 4.35**	89.94 ± 2.30	**93.04 ± 2.34**	**86.99 ± 3.95**
**Patch size**	**64 × 64 × 32**				**96 × 96 × 32**			
**Network**	**Pre (%)**	**Re (%)**	**Di (%)**	**IoU (%)**	**Pre (%)**	**Re (%)**	**Di (%)**	**IoU (%)**
VoxResnet [59]	93.89 ± 2.03	90.65 ± 2.51	92.21 ± 1.73	85.54 ± 2.99	93.89 ± 2.03	90.65 ± 2.51	92.21 ± 1.73	85.54 ± 2.99
Resnet (deep = 18) [60]	82.97 ± 2.21	86.25 ± 3.08	85.19 ± 2.72	74.20 ± 4.21	82.91 ± 3.27	85.00 ± 4.21	84.99 ± 3.10	73.90 ± 5.13
U-Net [13]	95.79 ± 3.83	90.00 ± 2.57	92.73 ± 2.4	86.45 ± 4.04	95.64 ± 3.73	89.21 ± 2.32	92.71 ± 2.65	86.41 ± 3.84
Attention U-Net [17]	96.36 ± 2.42	88.99 ± 3.70	92.8 ± 2.28	86.57 ± 3.72	94.12 ± 5.73	90.69 ± 2.65	92.82 ± 3.71	86.60 ± 5.58
Attention U-Net (MSFA)	96.74 ± 1.10	90.11 ± 2.81	93.15 ± 2.05	87.18 ± 3.50	**96.27 ± 1.98**	89.76 ± 3.47	93.00 ± 1.37	86.92 ± 3.16
Rattention U-Net [50]	93.61 ± 5.98	**90.65 ± 2.95**	91.99 ± 3.92	85.17 ± 6.02	95.22 ± 3.66	90.65 ± 2.21	92.09 ± 2.30	85.34 ± 3.39
Rattention U-Net (MSFA)	96.12 ± 1.96	89.73 ± 3.36	93.14 ± 2.23	87.16 ± 3.75	96.04 ± 1.53	89.71 ± 3.61	93.01 ± 1.70	86.93 ± 3.49
UARAI (Ours)	**96.90 ± 1.06**	90.62 ± 5.03	**93.20 ± 3.29**	**87.27 ± 4.85**	93.20 ± 4.85	**91.55 ± 2.98**	**93.05 ± 2.39**	**87.00 ± 3.52**
**Patch size**	**128 × 128 × 32**							
**Network**	**Pre (%)**	**Re (%)**	**Di (%)**	**IoU (%)**				
VoxResnet [59]	93.89 ± 2.03	90.65 ± 2.51	92.21 ± 1.73	85.54 ± 2.99				
Resnet (deep = 18) [60]	81.90 ± 2.13	84.27 ± 3.58	84.46 ± 3.25	73.10 ± 3.24				
U-Net [13]	96.42 ± 1.25	88.34 ± 4.20	92.63 ± 2.62	86.27 ± 4.28				
Attention U-Net [17]	96.92 ± 1.90	89.15 ± 3.10	92.84 ± 2.01	86.64 ± 3.44				
Attention U-Net (MSFA)	97.00 ± 1.37	**89.54 ± 3.32**	92.74 ± 2.33	86.46 ± 3.87				
Rattention U-Net [50]	**97.07 ± 1.22**	86.40 ± 4.40	91.56 ± 2.61	84.43 ± 4.30				
Rattention U-Net (MSFA)	97.06 ± 0.58	87.79 ± 6.28	92.22 ± 3.80	85.56 ± 6.02				
UARAI (Ours)	97.07 ± 1.77	88.87 ± 3.65	**93.09 ± 2.04**	**87.07 ± 3.46**				

**Table 5:** The results of the ablation experiment on MSFA, where the best results are highlighted in bold red.

	Vessels Dataset				Airways Dataset			
Network	Pre (%)	Re (%)	Di (%)	IoU (%)	Pre (%)	Re (%)	Di (%)	IoU (%)
Baseline	91.60 ± 1.92	86.49 ± 1.03	88.95 ± 0.89	80.10 ± 1.44	96.34 ± 0.65	89.72 ± 3.15	93.25 ± 1.84	87.35 ± 3.15
Baseline + MSFA	91.88 ± 1.74	87.01 ± 1.62	89.07 ± 0.74	80.29 ± 1.22	96.68 ± 0.91	89.49 ± 3.33	93.28 ± 2.03	87.41 ± 3.45
Baseline + Att	89.98 ± 1.35	**88.48 ± 1.52**	88.17 ± 0.45	78.84 ± 1.05	97.04 ± 0.61	89.86 ± 3.72	92.87 ± 2.25	86.69 ± 3.68
Baseline + MSFA + Att	91.90 ± 1.24	86.36 ± 1.47	89.03 ± 0.60	80.23 ± 0.98	97.06 ± 1.37	89.99 ± 2.17	93.24 ± 1.76	87.34 ± 3.03
Baseline + Ra	90.17 ± 1.39	88.30 ± 1.23	89.21 ± 0.60	80.52 ± 0.99	95.35 ± 5.21	**90.55 ± 2.97**	92.80 ± 3.51	86.57 ± 5.50
Baseline + MSFA + Ra	92.60 ± 1.32	86.82 ± 1.35	89.60 ± 0.69	81.16 ± 1.14	96.34 ± 0.78	90.54 ± 5.03	93.27 ± 3.31	87.38 ± 4.89
Baseline + MSFA + Ra + Icp	**93.89 ± 1.22**	87.27 ± 2.15	**90.31 ± 0.82**	**82.33 ± 1.37**	**97.41 ± 0.56**	89.67 ± 3.37	**93.34 ± 1.98**	**87.51 ± 3.34**

**Table 6:** The results of the ablation experiment on ‘Ra + Icp’, where the best results are highlighted in bold red.

	Vessels Dataset				Airways Dataset			
Network	Pre (%)	Re (%)	Di (%)	IoU (%)	Pre (%)	Re (%)	Di (%)	IoU (%)
Baseline + MSFA	91.88 ± 1.74	87.01 ± 1.62	89.07 ± 0.74	80.29 ± 1.22	96.68 ± 0.91	89.49 ± 3.33	93.28 ± 2.03	87.41 ± 3.45
Baseline + MSFA + Att	91.90 ± 1.24	86.36 ± 1.47	89.03 ± 0.60	80.52 ± 0.98	97.06 ± 1.37	89.99 ± 2.17	93.24 ± 1.76	87.34 ± 3.03
Baseline + MSFA + RA + Icp	**93.89 ± 1.22**	**87.27 ± 2.15**	**90.31 ± 0.82**	**82.33 ± 1.37**	**97.41 ± 0.56**	89.67 ± 3.37	**93.34 ± 1.98**	**87.51 ± 3.34**

## Data Availability

Some data comes from public datasets: https://data.kitware.com/#collection/591086ee8d777f16d01e0724, accessed on 20 September 2021, and private data is not applicable.

## References

[1] Longde W. (2017). Summary of 2016 Report on Prevention and Treatment of Stroke in China. Chin. J. Cerebrovasc. Dis..

[2] Wang C., Xu J., Yang L., Xu Y., Zhang X., Bai C., Kang J., Ran P., Shen H., Wen F. (2018). Prevalence and risk factors of chronic obstructive pulmonary disease in China (the China Pulmonary Health [CPH] study): A national cross-sectional study. Lancet N. Am. Ed..

[3] Palágyi K., Tschirren J., Hoffman E.A., Sonka M. (2006). Quantitative analysis of pulmonary airway tree structures. Comput. Biol. Med..

[4] Society N., Chinese Medical Doctor Association (2022). Expert consensus on the clinical practice of neonatal brain magnetic resonance imaging. Chin. J. Contemp. Pediatr..

[5] Sanchesa P., Meyer C., Vigon V., Naegel B. (2019). Cerebrovascular network segmentation of MRA images with deep learning. Proceedings of the 2019 IEEE 16th International Symposium on Biomedical Imaging (ISBI 2019).

[6] Fan S., Bian Y., Chen H., Kang Y., Yang Q., Tan T. (2020). Unsupervised cerebrovascular segmentation of TOF-MRA images based on deep neural network and hidden markov random field model. Front. Neuroinformatics.

[7] Zhao F., Chen Y., Hou Y., He X. (2019). Segmentation of blood vessels using rule-based and machine-learning-based methods: A review. Multimed. Syst..

[8] Hilbert A., Madai V., Akay E., Aydin O., Behland J., Sobesky J., Galinovic I., Khalil A., Taha A., Wuerfel J. (2020). BRAVE-NET: Fully automated arterial brain vessel segmentation in patients with cerebrovascular disease. Front. Artif. Intell..

[9] Kuo W., de Bruijne M., Petersen J., Nasserinejad K., Ozturk H., Chen Y., Perez-Rovira A., Tiddens H.A. (2017). Diagnosis of bronchiectasis and airway wall thickening in children with cystic fibrosis: Objective airway-artery quantification. Eur. Radiol..

[10] Tschirren J., Yavarna T., Reinhardt J. Airway segmentation framework for clinical environments. Proceedings of the 2nd International Workshop Pulmonary Image Analysis.

[11] Guo X., Xiao R., Lu Y., Chen C., Yan F., Zhou K., He W., Wang Z. (2021). Cerebrovascular segmentation from TOF-MRA based on multiple-U-net with focal loss function. Comput. Methods Programs Biomed..

[12] Ronneberger O., Fischer P., Brox T. U-net: Convolutional networks for biomedical image segmentation. Proceedings of the International Conference on Medical Image Computing and Computer-Assisted Intervention.

[13] Çiçek Ö., Abdulkadir A., Lienkamp S., Brox T., Ronneberger O. (2016). 3D U-Net: Learning dense volumetric segmentation from sparse annotation. Proceedings of the Medical Image Computing and Computer-Assisted Intervention–MICCAI 2016: 19th International Conference.

[14] Tetteh G., Efremov V., Forkert N., Schneider M., Kirschke J., Weber B., Zimmer C., Piraud M., Menze B. (2020). Deepvesselnet: Vessel segmentation, centerline prediction, and bifurcation detection in 3-d angiographic volumes. Front. Neurosci..

[15] Livne M., Rieger J., Aydin O., Taha A., Akay E., Kossen T., Sobesky J., Kelleher J., Hildebrand K., Frey D. (2019). A U-Net deep learning framework for high performance vessel segmentation in patients with cerebrovascular disease. Front. Neurosci..

[16] Lee K., Sunwoo L., Kim T., Lee K. (2021). Spider U-Net: Incorporating inter-slice connectivity using LSTM for 3D blood vessel segmentation. Appl. Sci..

[17] Oktay O., Schlemper J., Folgoc L., Lee M.L., Heinrich M., Misawa K., Mori K., McDonagh S., Hammerla Y., Kainz B. (2018). Attention u-net: Learning where to look for the pancreas. arXiv.

[18] Lo P., Van Ginneken B., Reinhardt J.M., Yavarna T., de Jong P.A., Irving B., Fetita C., Ortner M., Pinho R., Sijbers J. (2012). Extraction of airways from CT (EXACT’09). IEEE Trans. Med. Imaging.

[19] Park J.W. (2005). Connectivity-based local adaptive thresholding for carotid artery segmentation using MRA images. Image Vis. Comput..

[20] Wang R., Li C., Wang J., Wei X., Li Y., Zhu Y., Zhang S. (2015). Threshold segmentation algorithm for automatic extraction of cerebral vessels from brain magnetic resonance angiography images. J. Neurosci. Methods.

[21] Chen P., Zou T., Chen J.Y., Gao Z., Xiong J. (2015). The application of improved pso algorithm in pmmw image ostu threshold segmentation. Applied Mechanics and Materials.

[22] Zhu Q., Jing L., Bi R. (2010). Exploration and improvement of Ostu threshold segmentation algorithm. Proceedings of the 2010 8th World Congress on Intelligent Control and Automation.

[23] Neumann J.O., Campos B., Younes B., Jakobs A., Unterberg A., Kiening K., Hubert A. (2019). Evaluation of three automatic brain vessel segmentation methods for stereotactical trajectory planning. Comput. Methods Programs Biomed..

[24] Rad A.E., Mohd Rahim M.S., Kolivand H., Amin I. (2017). Morphological region-based initial contour algorithm for level set methods in image segmentation. Multimed. Tools Appl..

[25] Frangi A.F., Niessen W., Vincken K., Viergever M. (1998). Multi-scale vessel enhancement filtering. Proceedings of the International Conference on Medical Image Computing and Computer-Assisted Intervention.

[26] Mori K., Hasegawa J., Toriwaki J., Anno H., Katada K. Recognition of bronchus in three-dimensional X-ray CT images with application to virtualized bronchoscopy system. Proceedings of the 13th International Conference on Pattern Recognition.

[27] Sonka M., Park W., Hoffman E. (1996). Rule-based detection of intrathoracic airway trees. IEEE Trans. Med. Imaging.

[28] Tschirren J., Hoffman E., McLennan G., Sonka M. (2005). Intrathoracic airway trees: Segmentation and airway morphology analysis from low-dose CT scans. IEEE Trans. Med. Imaging.

[29] Duan H.H., Gong J., Sun X.W., Nie S. (2020). Region growing algorithm combined with morphology and skeleton analysis for segmenting airway tree in CT images. J. X-Ray Sci. Technol..

[30] Bhargavi K., Jyothi S. (2014). A survey on threshold based segmentation technique in image processing. Int. J. Innov. Res. Dev..

[31] Litjens G., Kooi T., Bejnordi B.E., Setio A., Ciompi F., Ghafoorian M., Laak J., Ginneken B., Sanchez C.I. (2017). A survey on deep learning in medical image analysis. Med. Image Anal..

[32] Ke Q., Zhang J., Wei W., Polap D., Wozniak M., Kosmider L., Damasevicius R. (2019). A neuro-heuristic approach for recognition of lung diseases from X-ray images. Expert Syst. Appl..

[33] Jaszcz A., Połap D., Damaševičius R. (2022). Lung x-ray image segmentation using heuristic red fox optimization algorithm. Sci. Program..

[34] Min Y., Nie S. Automatic Segmentation of Cerebrovascular Based on Deep Learning. Proceedings of the 2021 3rd International Conference on Artificial Intelligence and Advanced Manufacture.

[35] Mou L., Zhao Y., Fu H., Liu Y., Cheng J., Zheng Y., Su P., Yang J., Chen L., Frangi A.F. (2021). CS2-Net: Deep learning segmentation of curvilinear structures in medical imaging. Med. Image Anal..

[36] Xia L., Zhang H., Wu Y., Song R., Ma Y., Mou L., Liu J., Xie Y., Ma M., Zhao Y. (2022). 3D vessel-like structure segmentation in medical images by an edge-reinforced network. Med. Image Anal..

[37] Chen Y., Jin D., Guo B., Bai X. (2022). Attention-Assisted Adversarial Model for Cerebrovascular Segmentation in 3D TOF-MRA Volumes. IEEE Trans. Med. Imaging.

[38] Banerjee S., Toumpanakis D., Dhara A.K., Wikstrom J., Strand R. (2022). Topology-Aware Learning for Volumetric Cerebrovascular Segmentation. Proceedings of the 2022 IEEE 19th International Symposium on Biomedical Imaging (ISBI).

[39] Jiang Y., Zhang Z., Qin S., Guo Y., Li Z., Cui S. APAUNet: Axis Projection Attention UNet for Small Target in 3D Medical Segmentation. Proceedings of the Asian Conference on Computer Vision.

[40] Meng Q., Roth H.R., Kitasaka T., Oda M., Ueno J., Mori K. (2017). Tracking and segmentation of the airways in chest CT using a fully convolutional network. Proceedings of the Medical Image Computing and Computer-Assisted Intervention—MICCAI 2017: 20th International Conference.

[41] Garcia-Uceda Juarez A., Tiddens H., de Bruijne M. Automatic airway segmentation in chest CT using convolutional neural networks. Proceedings of the Image Analysis for Moving Organ, Breast, and Thoracic Images: Third International Workshop, RAMBO 2018.

[42] Garcia-Uceda Juarez A., Selvan R., Saghir Z., de Bruijne M. A joint 3D UNet-graph neural network-based method for airway segmentation from chest CTs. Proceedings of the Machine Learning in Medical Imaging: 10th International Workshop, MLMI 2019, Held in Conjunction with MICCAI 2019.

[43] Wang C., Hayashi Y., Oda M., Itoh H., Kitasaka T., Frangi A.F., Mori K. Tubular structure segmentation using spatial fully connected network with radial distance loss for 3D medical images. Proceedings of the Medical Image Computing and Computer Assisted Intervention–MICCAI 2019: 22nd International Conference.

[44] Tan W., Liu P., Li X., Xu S., Chen Y., Yang J. (2022). Segmentation of lung airways based on deep learning methods. IET Image Process..

[45] Lin T.Y., Dollár P., Girshick R., He K., Hariharan B., Belongie S. Feature pyramid networks for object detection. Proceedings of the IEEE Conference on Computer Vision and Pattern Recognition.

[46] He K., Zhang X., Ren S., Sun J. (2015). Spatial pyramid pooling in deep convolutional networks for visual recognition. IEEE Trans. Pattern Anal. Mach. Intell..

[47] Zhao H., Shi J., Qi X., Wang X., Jia J. Pyramid scene parsing network. Proceedings of the IEEE Conference on Computer Vision and Pattern Recognition.

[48] Chen L.C., Papandreou G., Kokkinos I., Murphy K., Yuille A. (2017). Deeplab: Semantic image segmentation with deep convolutional nets, atrous convolution, and fully connected crfs. IEEE Trans. Pattern Anal. Mach. Intell..

[49] Hu J., Shen L., Sun G. Squeeze-and-excitation networks. Proceedings of the IEEE Conference on Computer Vision and Pattern Recognition.

[50] Fan D.P., Ji G.P., Zhou T., Chen G., Fu H., Shen J., Shao L. (2020). Pranet: Parallel reverse attention network for polyp segmentation. Proceedings of the Medical Image Computing and Computer Assisted Intervention–MICCAI 2020: 23rd International Conference.

[51] Bullitt E., Zeng D., Gerig G., Aylward S., Joshi S., Smith J.K., Lin W.L., Ewend M.G. (2005). Vessel tortuosity and brain tumor malignancy: A blinded study1. Acad. Radiol..

[52] Palumbo O., Dera D., Bouaynaya N.C., Fathallah-Shaykh H. (2018). Inverted cone convolutional neural network for deboning MRIs. Proceedings of the 2018 International Joint Conference on Neural Networks (IJCNN).

[53] Shorten C., Khoshgoftaar T.M. (2019). A survey on image data augmentation for deep learning. J. Big Data.

[54] Hesamian M.H., Jia W., He X., Kennedy P. (2019). Deep learning techniques for medical image segmentation: Achievements and challenges. J. Digit. Imaging.

[55] Biswas M., Kuppili V., Saba L., Edla D.R., Suri H.S., Cuadrado-Godia E., Laird J.R., Marinhoe R.T., Sanches J.M., Nicolaides A. (2019). State-of-the-art review on deep learning in medical imaging. Front. Biosci.—Landmark.

[56] Cao H., Wang Y., Chen J., Jiang D., Zhang X., Tian Q., Wang M. (2022). Swin-unet: Unet-like pure transformer for medical image segmentation. Proceedings of the Computer Vision–ECCV 2022 Workshops.

[57] Wang X., Zhanshan L., Yingda L. (2022). Medical image segmentation based on multi-scale context-aware and semantic adaptor. J. Jilin Univ. (Eng. Technol. Ed.).

[58] Liu S., Qi L., Qin H., Shi J., Jia J. Path aggregation network for instance segmentation. Proceedings of the IEEE Conference on Computer Vision and Pattern Recognition.

[59] Chen H., Dou Q., Yu L., Qin J., Heng P. (2018). VoxResNet: Deep voxelwise residual networks for brain segmentation from 3D MR images. NeuroImage.

[60] He K., Zhang X., Ren S., Sun J. Deep residual learning for image recognition. Proceedings of the IEEE Conference on Computer Vision and Pattern Recognition.

